# Additions to *Didymosphaeriaceae*: two novel species of *Chromolaenicola crataegicola* and *Paraphaeosphaeria fulva* with notes on antibacterial and antifungal activities

**DOI:** 10.3897/mycokeys.137.197035

**Published:** 2026-07-20

**Authors:** Wen-Xin Su, Ranagul Tieliwadi, Bo Zhang, Xiao-Hui Yuan, Xiao Li

**Affiliations:** 1 Sanjiang Laboratory, Changchun, Jilin Province, 130118, China Internationally Cooperative Research Center of China for New Germplasm Breeding of Edible Mushroom, Jilin Agricultural University Changchun China; 2 Joint International Research Laboratory of Modern Agricultural Technology, Ministry of Education, Jilin Agricultural University, Changchun, 130118, China Joint International Research Laboratory of Modern Agricultural Technology, Ministry of Education, Jilin Agricultural University Changchun China; 3 Internationally Cooperative Research Center of China for New Germplasm Breeding of Edible Mushroom, Jilin Agricultural University, Changchun 130118, China Jilin Provincial Straw-Edible Mushroom Industry Science and Technology Innovation Center, Jilin Agricultural University Changchun China; 4 Jilin Provincial Straw-Edible Mushroom Industry Science and Technology Innovation Center, Jilin Agricultural University, Changchun, 130118, China Sanjiang Laboratory Changchun China

**Keywords:** *
Didymosphaeriaceae
*, molecular phylogeny, new species, secondary metabolites, taxonomy

## Abstract

*Didymosphaeriaceae* is an important family within *Pleosporales*, widely distributed globally as saprophytes, pathogens and endophytes on plants, possessing potential for agricultural and medicinal applications. Due to the environmental risks posed by chemical pesticides, plant-associated microorganisms have become an important resource for developing eco-friendly biopesticides. During a study of *Didymosphaeriaceae* fungi with woody litter from northeast China, decaying wood tissue samples were collected and examined. Through morphological and molecular phylogenetic analysis (ITS, SSU, LSU, *tef*1-*α* and *rpb*2), we described two novel species, *Chromolaenicola crataegicola* and *Paraphaeosphaeria fulva* and preliminarily assessed the antibacterial and antifungal activities of their secondary metabolites. This research further enriches the diversity of plant-associated ascomycetes in northeast China and highlights their potential strains for the development of biopesticides.

## Introduction

*Didymosphaeriaceae* fungi exist as endophytes, saprophytes and pathogens and have been associated with diseases in animals, plants and humans (Verkley et al. 2014; Hongsanan et al. 2020; Dissanayake et al. 2021; Ren et al. 2022; Phookamsak et al. 2024; Chen et al. 2025; Tennakoon et al. 2025). Exhibiting a wide global distribution, the family has been reported across diverse habitats and hosts (Aptroot 1995; Ariyawansa et al. 2014; Thambugala et al. 2017; Phookamsak et al. 2019; Maharachchikumbura et al. 2021). The family currently includes 39 genera (Hyde et al. 2024); however, the lack of molecular data for many type species has left phylogenetic relationships amongst several genera, such as *Paraconiothyrium* and *Kalmusia*, unresolved (Khodaei et al. 2019). Therefore, multi-locus sequence data (ITS, LSU, SSU, *tef*1-*α*, *rpb*2) are essential for accurate genus placement and species identification within *Didymosphaeriaceae* (Khodaei et al. 2019; Samarakoon et al. 2020).

Mapook et al. (2020) introduced *Chromolaenicola* into *Didymosphaeriaceae* with *C. nanensis* as the type species. Currently, *Chromolaenicola* encompasses 12 species records (Species Fungorum: https://www.speciesfungorum.org; accession date: 5 July 2026), primarily from China and Thailand. The genus exhibits substantial host diversity, including plant hosts, such as *Ananas comosus*, *Bidens pilosa*, *Chromolaena odorata*, *Clematis subumbellata*, *Citrus australasica*, *Leucaena* sp. and *Sapindus rarak* (Jayasiri et al. 2019; Mapook et al. 2020; Phukhamsakda et al. 2020; Htet et al. 2024; Tian et al. 2024; Wanasinghe et al. 2025). This extensive host range suggests remarkable ecological adaptability across diverse niches and further sampling efforts are likely to reveal additional species within the genus.

Eriksson (1967) introduced *Paraphaeosphaeria*, with *P. michotii* as the type species. The genus currently comprises 53 species (Hyde et al. 2024; Du et al. 2025; Species Fungorum: https://www.speciesfungorum.org; accession date: 5 July 2026). Phylogenetic studies have recovered *Paraphaeosphaeria* as a well-supported lineage within *Didymosphaeriaceae* (Ariyawansa et al. 2014). The sexual morph of *Paraphaeosphaeria* is characterised by immersed ascomata, bitunicate asci and multi-septate ascospores (Wong et al. 2000; Ariyawansa et al. 2014). The asexual morph is coelomycetous with coniothyrium-like or phoma-like spores (Verkley et al. 2014; Wanasinghe et al. 2018; Hongsanan et al. 2020). Species of *Paraphaeosphaeria* are typically endophytic, saprobic and intestinal fungi, occurring on various hosts. For instance, *P. angularis* has been isolated from leaves of *Saccharum officinarum* in India (Malarvizhi et al. 2022); *P. michotii* is commonly found on decaying stems and leaves of *Carex* spp. (*Cyperaceae*) and *Juncus* spp. (*Juncaceae*) (Wong et al. 2000); and *P. verruculosa* shows remarkable adaptability, having been isolated from wood logs of *Pinus radiata* and also reported from animal intestinal tracts (Verkley et al. 2014; Hu et al. 2020; Sun et al. 2020).

*Didymosphaeriaceae* fungi are increasingly recognised for their ecological versatility and bioactivity. Vidal et al. (2023) isolated the marine fungus *Chromolaenicola* sp. (HL-114-33-R04) from the *Rhodophyta* red algae *Peyssonnelia* and demonstrated that it could play a role in cancer treatment. *Paraphaeosphaeria* species have been shown to produce bioactive secondary metabolites with antibacterial, antifungal and herbicidal activities (Shao 2015; Hu et al. 2020). For instance, *P. verruculosa* produces antifungal compounds effective against plant pathogens, such as *Colletotrichum gloeosporioides*, *Didymella glomerata* and *Nigrospora oryzae* (Hu et al. 2020). Moreover, *Paraphaeosphaeria* sp. (XZD2-1) exhibits cytotoxic and antibacterial properties, further illustrating the biological activity potential of the genus (Sun et al. 2020).

This study aims to define and formally describe newly-discovered species, based on morphological characters and multi-locus phylogenetic analysis of ITS, SSU, LSU, *tef*1-*α* and *rpb*2 sequence data. Simultaneously, we conducted preliminary investigations into their biological activities to evaluate their potential as sources of novel strains for next-generation biopesticide development.

## Materials and methods

### Sample collection, morphological study and isolation

Specimens were collected from Changchun City, Jilin Province (43°53'49"N, 125°19'34"E). After labelling, the samples were placed in sealed bags and returned to the laboratory for subsequent analysis. Using a stereomicroscope, the ascomata or pycnidia were cut with a sterile blade and transferred on to a glass slide containing a drop of sterile water using a sterile needle to prepare a spore suspension. The suspension was aspirated and evenly spread on to PDA and kept at 25 °C in the dark for 24 h. Germinated ascospores were transferred aseptically to PDA and grown at 25 °C for 2 weeks. All the micro-morphological characters (conidiomata, pycnidial wall, conidiogenous cells and conidia) were observed using a Zeiss Image A2 (Zeiss, Oberkochen, Germany) and a microscope, equipped with Leica DFC450C (Leica, Heidelberg, Germany). Under the microscope, the structure and size of microcharacters were measured using ZEN 3.4 (Blue Edition) software (Zeiss, Germany). The photos were edited using Adobe Photoshop CC2020 (Adobe Systems, USA). Type specimens were deposited in the Herbarium of Mycology, Jilin Agricultural University (HMJAU). Pure cultures were deposited in the Engineering Research Center of Edible and Medicinal Fungi, Ministry of Education Culture Collection (EMFCC), Changchun, China. New taxa were registered in MycoBank (https://www.mycobank.org/).

### DNA extraction, PCR amplification and sequencing

Genomic DNA was extracted from pure cultures according to the manufacturer’s protocol using the NuClean PlantGen DNA extraction kit (CWBIO, China). The internal transcribed spacer region of ribosomal DNA (ITS) was amplified using primers ITS5 and ITS4 (White et al. 1990); nuclear small subunit rDNA (18S, SSU) using primers NS1 and NS4; the large subunit (LSU) of ribosomal DNA using primers LR0R and LR5 (Vilgalys and Hester 1990); the RNA polymerase II second-largest subunit (*rpb*2) using primers fRPB2-5f and fRPB2-7cr (Voglmayr et al. 2016); and the translation elongation factor 1-alpha (*tef*1-*α*) using primers EF1-983F and EF1-2218R (Rehner and Buckley 2005). PCR amplification of the ITS, LSU and SSU regions was performed under identical conditions, involving 5 min of initial denaturation at 94 °C, followed by 35 cycles (each cycle comprising 30 seconds of denaturation at 94 °C, 45 s of annealing at 53 °C and 90 s of extension at 72 °C), with a final extension at 72 °C for 10 min. For *rpb*2 and *tef*1-*α*, the annealing was performed at 56 °C for 30 s, while all other steps remained unchanged.

The amplification reactions were conducted using 20 μl PCR mixtures comprising 9 μl of ddH_2_O, 10 μl of 2× EsTaq MasterMix (Dye), 0.4 μl of DNA template and 0.3 μl of each primer at a concentration of 2 μmol/μl. All PCR products were visualised through electrophoresis on a 1% agarose gel. Subsequently, the PCR products were sequenced by Sangon Biotech (Shanghai) Co., Ltd., China.

### Phylogenetic analysis

The newly-obtained sequences were subjected to BLASTn analysis against GenBank (http://blast.ncbi.nlm.nih.gov/) to identify the most closely-related sequences to those newly obtained. Reference sequence data were obtained from GenBank, based on recent literature (Table 1). All obtained sequence data were aligned using MAFFT v. 7.0 (https://mafft.cbrc.jp/alignment/server/) (Katoh et al. 2019), with manual correction of ambiguous nucleotide sites in AliView as needed and removal of redundant sequences outside the primer binding regions (Larsson 2014). The sequence datasets assembled were processed in SequenceMatrix v.1.7.8 (Vaidya et al. 2011). Phylogenetic trees were constructed using the Maximum Likelihood (ML) method and the Bayesian Inference (BI) method, with detailed analyses performed on the XSEDE platform of the CIPRES portal (http://www.phylo.org/portal2/) using RAxML-HPC2 v.8.2.12 and MrBayes v. 3.2.6.

**Table 1.** Names, strain numbers and corresponding GenBank accession numbers of taxa used in this study.

**Taxon**	**Strain Number**	**GenBank Accession Numbers**					**Country**
		**ITS**	**SSU**	**LSU**	***tef*1-α**	***rpb*2**	
*Agrorhizomyces patris*	BBT16	PP264950	PP264972	PP265004	PP273252	–	Hungary: Martonvásár
*Agrorhizomyces patris*	CBS 151043=BBT01T	NR 197907	PP264967	NG_243992	PP273247	–	Hungary: Martonvásár
*Alloconiothyrium aptrootii*	CBS 980.95 T	JX496121	–	JX496234	–	–	Papua New Guinea, Central Province
*Alloconiothyrium aptrootii*	CBS 981.95	JX496122	–	JX496235	–	–	Papua New Guinea: Central Province
*Austropleospora keteleeriae*	MFLUCC 18-1551=KUMCC 18-0217 T	NR_163349	MK347910	NG_070075	MK360045	MK434909	China: Yunnan
*Austropleospora ochracea*	KUMCC 20-0020 T	MT799859	NG_081371	MT799860	MT872714	–	China: Guizhou
*Periconia didymospora*	MFLU 15-0057	KP761733	KP761737	KP761730	KP761727	KP761720	Thailand
*Periconia didymospora*	MFLU 15-0058	KP761734	KP761738	KP761731	KP761728	KP761721	Thailand
*Bimuria novae-zelandiae*	CBS 107.79=AFTOL ID 931 T	NR_159620	NG_061017	NG_058623	Genome	Genome	–
*Bimuria omanensis*	SQUCC 15280 T	MT274326	_	MT271820	MT279046	–	Oman: Jebel Akhdar
*Byssothecium circinans outgroup*	CBS 675.92	OM337536	GU205235	GU205217	Genome	Genome	–
*Chlamydosphaeromyces glomeratus*	YNE00904	NMDCN0007RIL	–	NMDCN0007RIR	NMDCN0007RJ8	NMDCN0007RJK	China: Yunnan
*Chlamydosphaeromyces glomeratus*	YNE00913	NMDCN0007RIM	–	NMDCN0007RIS	NMDCN0007RJ9	NMDCN0007RJL	China: Yunnan
*Chromolaenicola ananasi*	MFLU23-0167	OR438340	NG_245293	OR438811	OR500305	-	Thailand
*Chromolaenicola chiangraiensis*	MFLUCC 17-1493 T	MN325017	MN325011	NG_070944	MN335650	MN335655	Thailand: Chiang Rai
*Chromolaenicola clematidis*	MFLUCC17-2075	MT310601	MT226671	MT214554	-	-	Thailand
*Chromolaenicola crataegicola*	EMFCC 0089	PZ096738	PZ096742	PZ096746	PZ213744	PZ213748	China
*Chromolaenicola crataegicola*	EMFCC 0090	PZ096739	PZ096743	PZ096747	PZ213745	PZ213749	China
*Chromolaenicola doitungensis*	MFLUCC 24-0545	PQ800251	PV072698	PV072605	PX739629	-	Thailand
*Chromolaenicola hongheensis*	KUNCC23-16762	PV742899	PV743008	PV742955	PV700634	PV700680	China
*Chromolaenicola hongheensis*	KUNCC23-16760	PV742900	PV743009	PV742956	PV700635	PV700681	China
*Chromolaenicola hydei*	UESTCC23-0437	PQ394027	-	PQ184678	PQ346470	-	China
*Chromolaenicola hydei*	CGMCC3.27710	PQ394062	-	PQ184707	PQ346508	-	China
*Chromolaenicola hydeiwikstroemiae*	UESTCC23-0436	PQ394030	-	PQ184681	PQ346473	PQ379939	China
*Chromolaenicola hydeiwikstroemiae*	CGMCC3.27711	PQ394063	-	PQ184708	PQ346509	PQ379993	China
*Chromolaenicola lampangensis*	MFLUCC17-1462T	MN325016	MN325010	MN325004	MN335649	MN335654	Thailand
*Chromolaenicola nanensis*	MFLUCC 17-1473 T	MN325015	MN325009	NG_070942	MN335648	MN335653	Thailand: Chiang Rai
*Chromolaenicola sapindi*	KUMCC21-0564	OP058967	OP059009	OP059058	OP135943	-	Thailand
*Chromolaenicola sapindi*	KUMCC21-0594	OP058968	OP059010	OP059059	OP135944	-	Thailand
*Chromolaenicola siamensis*	MFLUCC17-2527	NR_163337	-	NG_066311	MK360048	MK434882	Thailand
*Chromolaenicola siamensis*	C329	MK347760	MK347866	MK347976	-	-	Thailand
*Chromolaenicola siamensis*	MFLU 24-0029	_	_	_	PP474192.1	PP474189.1	_
*Chromolaenicola thailandensis*	MFLUCC17-1510	MN325018	MN325012	MN325006	MN335651	-	Thailand
*Chromolaenicola thailandensis*	MFLUCC1-1475	MN325019	MN325013	MN325007	MN335652	MN335656	Thailand
*Coniothyrium juniperi*	CBS 610.72 T	MH860594	–	MH872291	–	–	–
*Cylindroaseptospora bennettiae*	BRIP 72408d	OP599619	-	OP598060	-	-	Australia
*Cylindroaseptospora leucaenae*	MFLUCC 17-2424=KUMCC 18 0226 T	NR_163333	MK347856	NG_066310	MK360047	–	Thailand: Chiang Rai
*Deniquelata barringtoniae*	MFLUCC 11-0422 T	NR_111779	JX254656	NG_042696	–	–	Thailand: Chiang Rai
*Deniquelata barringtoniae*	MFLUCC 16-0271=KUMCC 16-0161	MH275059	_	MH260291	MH412766	MH412753	Thailand: Prachuap Khiri Khan
*Deniquelata hypolithi*	CBS 146988	MZ064429	-	MZ064486	-	-	Namibia
*Deniquelata macphersoniae*	BRIP 72538b	OP599620	-	OP598061	-	-	Australia
*Deniquelata quercina*	1050 SAB	MT820404	-	MT808605	-	-	Brazil
*Deniquelata quercina*	ABRIICC 10068=20SA T	MH316153	MH316155	MH316157	–	–	Iran: Ilam
*Dictyoarthrinium hydei*	SQUCC 13296 T	MW077145	MW077161	–	MW075771	–	–
*Dictyoarthrinium musae*	MFLUCC 20-0105 T	MT482323	MT482326	MT482320	–	–	–
*Didymocrea sadasivanii*	CBS 438.65 T	MH858658	DQ384066	DQ384103	–	–	–
*Didymosphaeria brasiliense*	CBS 100299 T	NR_163552	AY642523	NG_070605	–	–	Brazil: Patrocinio-Minas Gerais
*Didymosphaeria rubi-ulmifolii*	MFLUCC 14-0023 T	–	NG 063557	KJ436586	–	–	Italy: Forlì-Cesena
*Didymosphaeria rubi-ulmifolii*	MFLUCC 14-0024 T	–	KJ436587	KJ436585	–	–	Italy: Forlì-Cesena
*Didymosphaeria variabile*	CBS 121163 STE U 6311 T	NR_137006	NG_064914	–	–	–	South Africa: Western Cape
*Didymosphaeria variabile*	CBS 121754=STE U 6313	JX496031	–	JX496144	–	–	South Africa: Western Cape
*Didymosphaeriaceae* sp.	NME00265	NMDCN0007RI7	–	NMDCN0007RIN	NMDCN0007RJ4	NMDCN0007RJH	China: Inner Mongolia
*Kalmusia araucariae*	CPC 37475	MT223805	-	MT223900	-	-	USA
*Kalmusia cordylines*	ZHKUCC 21-0092	OL352082	-	NG 088312	-	-	China
*Kalmusia ebuli*	CBS 123120 T	KF796674	-	JN644073	–	–	–
*Kalmusia longispora*	CBS 824.84 T	NR_153979	–	MH873526	–	–	Manitoba
*Kalmusia variispora*	CBS 121517	MH863113	–	MH874668	–	–	–
*Kalmusibambusa triseptata*	MFLUCC 13-0232=KUMCC 16-0183 T	KY682697	KY682696	KY682695	–	–	Thailand: Chiang Rai
*Karstenula rhodostoma*	CBS 690.94	OM337539	GU296154	GU301821	Genome	Genome	Sweden
*Karstenula rhodostoma*	CBS 691.94	_	AB797241	AB807531	AB808506	–	–
*Laburnicola centaureae*	MFLUCC 13-0601 T	KX274239	KU743193	KU743192	KU743212	–	Italy: Forlì-Cesena
*Laburnicola hawksworthii*	MFLUCC 13-0602 T	KU743194	KU743196	KU743195	–	–	Italy: Forlì-Cesena
*Laburnicola muriformis*	MFLUCC 16-0290 T	KU743197	KU743199	KU743198	KU743213	–	Italy: Forlì-Cesena
*Laburnicola radiciphila*	F6B1=DSM112862 T	NR_187029	ON876670	ON870566	ON892832	–	Hungary: Kiskunság
*Laburnicola rhizohalophila*	CGMCC 8756=JP R 44 T	KJ125522	–	KJ125523	KJ125525	KJ125524	China: Shandong
*Letendraea cordylinicola*	MFLUCC 11-0148 T	NR_154118	NG_068362	NG_059530	–	–	Thailand: Mae Jai
*Letendraea eurotioides*	CBS 212.31	–	–	AY787935	–	–	–
*Massarina eburnea outgroup*	CBS 473.64	OM337528	GU296170	GU301840	GU349040	GU371732	–
*Montagnula aquatica*	YRS 2022a	OP605992	OP600504	OP605986	-	-	Thailand
*Montagnula chromolaenicola*	MFLUCC 17-1469 T	NR_168866	NG_070157	NG_070948	MT235773	MT235809	Thailand: Mae Hong Son
*Montagnula chromolaenicola*	YNE01157	NMDCN0007RIK	–	NMDCN0007RJ0	NMDCN0007RJD	NMDCN0007RJP	China: Yunnan
*Montagnula graminicola*	MFLUCC 13-0352 T	KM658314	KM658316	KM658315	–	–	Italy: Forlì-Cesena
*Montagnula guiyangensis*	HKAS 124556	OP605989	OP600500	OP600484	-	-	China
*Montagnula puerensis*	KUMCC 20-0225	MW567739	MW575864	MW575866	MW573959	-	China
*Montagnula saikhuensis*	MFLUCC 16-0315 T	KU743209	KU743211	KU743210	–	–	Thailand: Prachuap Khiri Khan
*Montagnula thailandica*	MFLUCC 17-1508 T	MT214352	NG_070158	NG_070949	MT235774	MT235810	Thailand: Chiang Mai
*Neokalmusia brevispora*	NBRC 106240 T	NR_154262	AB524460	AB524601	AB539113	AB539100	Japan: Hokkaido
*Neokalmusia deguarnae*	BRIP 75884a T	NR_198784	–	PP707922	–	–	Australia: Queensland
*Neokalmusia jonahhulmei*	KUMCC 21-0818 T	NR_182587	NG_148908	ON007039	ON009133	ON009137	China: Yunnan
*Neokalmusia kunmingensis*	KUMCC 18-0120 T	MK079886	MK079887	MK079889	MK070172	–	China: Yunnan
*Neptunomyces aureus*	CMG 10A=MUM 19-38 T	MK912119	–	–	MK947998	–	Portugal: Ria de Aveiro
*Neptunomyces juncicola*	CBS 150790=CPC 45436	NR_197932	–	NG_244054	PP780627	–	Netherlands: North Holland
*Neptunomyces litoralis*	BRIP 75555a	NR_189984	–	NG_242141	–	–	Australia: New South Wales
*Neptunomyces yunnanensis*	YNE00523	NMDCN0007RIH	–	NMDCN0007RIP	NMDCN0007RJ6	NMDCN0007RJI	China: Yunnan
*Paracamarosporium fagi*	CPC 24890=CBS 140008	NR_154318	–	NG_070630	–	–	–
*Paracamarosporium hawaiiense*	CBS 120025	JX496027	EU295655	JX496140	–	–	South Africa: Western Cape
*Paracamarosporium psoraleae*	CPC 21632=CBS 136628 T	KF777143	–	KF777199	–	–	South Africa: Western Cape
*Paraconiothyrium ajrekarii*	NFCCI 4810	MT372906	-	MT372905	-	-	India
*Paraconiothyrium archidendri*	CBS168 77	MH861045	-	MH872813	-	-	Myanmar
*Paraconiothyrium bishopiae*	BRIP 72437b	NR_182624	–	OP598066	–	–	_
*Paraconiothyrium bishopiae*	YNE00964	NMDCN0007RIA	–	NMDCN0007RIT	NMDCN0007RJA	NMDCN0007RJM	China: Yunnan
*Paraconiothyrium cyclothyrioides*	CBS 972 95	JX496119	AY642524	JX496232	–	–	Papua New Guinea: Central Province
*Paraconiothyrium estuarinum*	CBS 109850=CCT6596	MH862842	AY642522	MH874432	–	LT854937	Brazil: São Paulo State
*Paraconiothyrium hakeae*	CBS 142521=CPC 27651	NR_171729	–	KY979809	–	KY979847	Australia: Western Australia
*Paraconiothyrium iridis*	CPC 36281=CBS 146036 T	NR_170059	–	NG_074423	–	MT223695	Ukraine: Chernihiv region
*Paraconiothyrium marshiae*	BRIP 75785a	PQ061115	–	PQ047740	–	–	–
*Paraconiothyrium thysanolaenae*	MFLUCC 10-0550 T	KP744453	KP753959	KP744496	–	–	Thailand: Chiang Mai
*Paraconiothyrium zingiberacearum*	YNE00613	NMDCN0007RI8	–	NMDCN0007RIQ	NMDCN0007RJ7	NMDCN0007RJJ	China: Yunnan
*Paraconiothyrium zingiberacearum*	YNE01332	NMDCN0007RI9	–	NMDCN0007RJ1	NMDCN0007RJE	NMDCN0007RJQ	China: Yunnan
*Paramassariosphaeria anthostomoides*	CBS 615.86	-	GU205246	MH873693	–	–	–
*Paramassariosphaeria clematidicola*	MFLUCC 16-0172 T	KU743206	KU743208	KU743207	–	–	Italy: Forlì-Cesena
*Paraphaeosphaeria angularis*	CBS 167.70	JX496047	-	MH871317	-	-	Brazil
*Paraphaeosphaeria arecacearum*	CBS 614.75	JX496100	-	MH872725	-	-	Ivory Coast
*Paraphaeosphaeria arecacearum*	CBS 158.75	JX496043	-	JX496156	-	-	Ivory Coast
*Paraphaeosphaeria brachiariae*	NCYUCC 19-0343	ON117289	ON117271	ON117307	-	-	Thailand
*Paraphaeosphaeria burbidgeae*	BRIP72477a	OP599630	-	OP598067	-	-	China
*Paraphaeosphaeria fulva*	EMFCC 0084	PZ096740	PZ096744	PZ096748	PZ213746	-	China
*Paraphaeosphaeria fulva*	EMFCC 0085	PZ096741	PZ096745	PZ096749	PZ213747	-	China
*Paraphaeosphaeria graminicola*	MFLUCC 15-0450	KX965729	-	KX954398	-	-	China
*Paraphaeosphaeria hydei*	CGMCC3.19317	MK329127	-	MK329032	MK336062	-	Australia
*Paraphaeosphaeria hydei*	LC12565	MK329128	-	MK329033	MK336063	-	Australia
*Paraphaeosphaeria hydeihelleniae*	CGMCC 3.27706	PQ394065	PQ184752	PQ184712	PQ346512	-	China
*Paraphaeosphaeria hydeihelleniae*	UESTCC 230429	PQ394033	PQ184744	PQ184686	PQ346477	-	China
*Paraphaeosphaeria inverness*	BRIP75560a	OR608745	-	OR602939	-	-	Italy
*Paraphaeosphaeria jaguarinae*	BRIP67031a	OR673889	-	-	-	-	Italy
*Paraphaeosphaeria jaguarinae*	HKAS146030	PV742904	PV743013	PV742960	PV700639	PV700684	Netherlands
*Paraphaeosphaeria kovalevskayae*	BRIp75811a	OR290130	-	OR288587	-	-	Netherlands
*Paraphaeosphaeria michotii*	MFLUCC 13-0349	KJ939279	KJ939285	KJ939282	-	KP998465	Italy
*Paraphaeosphaeria michotii*	CBS 340.86	JX496079	–	JX496192	–	–	–
*Paraphaeosphaeria minitans*	CBS 859.71	JX496116	–	JX496229	–	–	USA: California
*Paraphaeosphaeria neglecta*	CBS 124077	JX496038	-	MH874871	-	-	Chile
*Paraphaeosphaeria neglecta*	CBS 124078 T	JX496039	–	JX496152	–	–	Italy: Latina
*Paraphaeosphaeria neglecta*	ZJE01696	NMDCN0007RIG	–	NMDCN0007RJ3	NMDCN0007RJG	NMDCN0007RJS	China: Zhejiang
*Paraphaeosphaeria oryzae*	MFLUCC24-0032	PQ374213	-	PQ374215	-	-	Sweden
*Paraphaeosphaeria parmeliae*	CBS131728	KP170654	-	KP170722	-	-	Belgium
*Paraphaeosphaeria pilleata*	SF 596	MT529872	-	-	-	-	Italy
*Paraphaeosphaeria pilleata*	SF 439	MT529715	-	-	-	-	Italy
*Paraphaeosphaeria rosae*	MFLUCC 17-2547	MG828935	MG829150	MG829044	MG829222	-	Sweden
*Paraphaeosphaeria rosae*	MFLUCC 17-2548	MG828936	MG829151	MG829045	-	-	Sweden
*Paraphaeosphaeria rosicola*	MFLUCC 15-0042	MG828938	MG829153	MG829047	-	-	Norway
*Paraphaeosphaeria sardoa*	CBS 501.71 T	JX496094	–	JX496207	–	–	Italy: Sardinia
*Paraphaeosphaeria sorghi*	COAD3305	NR_190246	-	-	-	-	France
*Paraphaeosphaeria spartii*	ZMQL3	MT446162	-	-	-	-	Brazil
*Paraphaeosphaeria spartii*	MFLU 14-C0810	KP711357	KP711367	KP711362	-	-	Australia
*Paraphaeosphaeria sporulosa*	CBS105.76	MH860965	-	MH872734	-	-	Germany
*Paraphaeosphaeria sporulosa*	CBS 391.86	JX496082	-	MH873666	-	-	Germany
*Paraphaeosphaeria traversiae*	BRIP75020a	OP903474	-	OP903496	-	-	Italy
*Paraphaeosphaeria verruculosa*	CBS 263.85	MH861879	-	MH873567	-	-	Chile
*Paraphaeosphaeria verruculosa*	CBS 682.84	MH861815	-	MH873507	-	-	Chile
*Paraphaeosphaeria verruculosa*	CBS 354.80	JX496080	-	JX496193	-	-	Chile
*Paraphaeosphaeria viciae*	MFLU 15-1231	KY379969	-	KY397947	-	-	Italy
*Paraphaeosphaeria viridescens*	CBS 854.73	MH860813	-	MH872545	-	-	Montenegro
*Paraphaeosphaeria xanthorrhoeae*	CBS 142164	KY979738	-	NG_070930	-	KY979845	South Africa
*Paraphaeosphaeria youngiae*	BRIP75896a	-	-	PQ431201	-	-	Australia
*Phaeodothis mori*	MFLUCC 18-1634	MN356454	MN356452	MN356450	MN364867	-	Taiwan
*Phaeodothis winteri*	CBS 182.58	_	GU296183	GU301857	DQ677917	–	–
*Pleoardoris graminearum*	DS304 T	_	MT707488	MT707514	–	–	USA: Texas
*Pleoardoris graminearum*	DS334	_	MT707489	MT707515	–	–	USA: Texas
*Proxiconiothyrium yunnanense*	YNE01575	NMDCN0007RIF	–	NMDCN0007RJ2	NMDCN0007RJF	NMDCN0007RJR	China: Yunnan
*Proxiconiothyrium yunnanense as Didymosphaeria* sp.	ARM 1124	PP277137	–	–	–	–	Brazil: Araripina
*Pseudocamarosporium corni*	MFLUCC 13-0541=ICMP 20369=GUCC 0010 T	KJ747048	KJ819946	KJ813279	–	–	Italy: Arezzo Province
*Pseudocamarosporium propinquum*	MFLUCC 13 0544=ICMP 20371=GUCC 0013 T	KJ747049	KJ819949	KJ813280	–	–	France: Rouen
*Pseudocamarosporium pteleae*	MFLUCC 17-0724 T	NR_157536	MG829166	MG829061	MG829233	–	USA: California
*Pseudocamarosporium ulmi-minoris*	MFLUCC 17-0671 T	NR_157537	MG829167	MG829062	–	–	USA: California
*Pseudodidymocyrtis lobariellae*	A.F 25130	MT153960	MT674676	MT153989	-	-	Bolivia
*Pseudopithomyces chartarum*	MUCL 15905	LK936375	–	LK936383	–	LK936439	Belgium
*Pseudopithomyces entadae*	MFLUCC 17-0917	-	-	NG_066305	MK360083	MK434899	Thailand
*Pseudopithomyces kunmingensis*	MFLUCC 17-0314=KUMCC 16-0222	MF173607	MF173606	MF173605	–	–	China: Yunnan
*Pseudopithomyces mori*	MFLUCC 18-1630=KUMCC 19-0130 T	MW063153	MW079343	MW063214	MW183777	–	Taiwan: Chiayi
*Spegazzinia bromeliacearum*	URM 8084 T	NR_191084	–	NG_242469	–	–	Brazil: Pernambuco state
*Spegazzinia radermacherae*	MFLUCC 17-2285=KUMCC 18-0297 T	MK347740	MK347848	MK347957	MK360088	MK434893	Thailand: Chiang Rai
*Spegazzinia tessarthra*	MAFF 243875	LC757459	–	–	–	–	Japan: Aomori
*Tremateia camporesii*	MFLU 19-2109	-	MN473050	MN473056	MN481602	–	China: Guizhou
*Tremateia chiangraiensis*	MFLUCC 17-1428 T	NR_168867	NG_070159	NG_068709	MT235775	MT235813	Thailand: Chiang Rai
*Verrucoconiothyrium nitidae*	CBS 119209=CMW 19988	EU552112	–	_	–	–	South Africa
*Vicosamyces venturisporus*	CDA1494	MF802825	–	MF802828	–	–	Brazil: Minas Gerais
*Vicosamyces venturisporus*	CDA1495	MF802826	–	MF802829	–	–	Brazil: Minas Gerais
*Xenocamarosporium acaciae*	CPC 24755=CBS 139895 T	NR_137982	–	NG_058163	–	–	Malaysia: Sabah
*Xenocamarosporium acaciae*	MFLUCC 17-2432	MK347766	MK347873	MK347983	MK360093	–	–

The best-fit nucleotide substitution models for individual and combined datasets were estimated using jModelTest v. 2.1.10 (Darriba et al. 2012) on the CIPRES web portal under the Akaike Information Criterion (AIC). The GTR+GAMMA model was selected as the best-fit model for all datasets. Maximum Likelihood (ML) phylogenetic analyses were then performed using RAxML-HPC v. 8.2.12 on the XSEDE platform (Stamatakis 2014), with a rapid bootstrapping algorithm of 1,000 bootstrap replicates, applying the GTR+G substitution model uniformly across all partitions.

For BI analysis, the programme ran four chains, with 5,000,000 iterations per chain. The tree was sampled once every 1200^th^ generation and the first 20% was discarded as burn-in. Bayesian posterior probabilities (BYPP) ≥ 0.90 were considered high statistical support (Ronquist and Huelsenbeck 2003). Trees were visualised in FigTree v. 1.4.4 and edited with Adobe Illustrator CS3 (Version 15.0.0, Adobe®, San Jose, CA) (Rambaut 2018). Newly-generated sequences have been submitted to GenBank (https://www.ncbi.nlm.nih.gov/).

### Preparation of crude extracts from secondary metabolites and antibacterial and antifungal tests

Pure culture of *Chromolaenicola crataegicola* (EMFCC 0084) and *Paraphaeosphaeria fulva* (EMFCC 0089) was inoculated into PDB liquid medium and fermented at 28 °C, 150 rpm for 14 days (Gao et al. 2021). After fermentation, the broth was extracted three times with equal volumes of ethyl acetate and the organic phases were combined. The extracts were dehydrated with anhydrous sodium sulphate, then concentrated by rotary evaporation at 40 °C until dry, yielding crude secondary metabolites, which were stored at -20 °C for later use.

The antibacterial and antifungal activities of crude extracts were evaluated using the mycelial growth rate and inhibition zone methods (Cui et al. 2022). The antifungal activity was tested against *Botrytis cinerea*, *Cladobotryum mycophilum* and *Trichoderma harzianum*. The crude extract was re-dissolved in methanol and a final concentration of 20 mg/ml was selected for the experiments. A mycelial plug (5 mm diameter) taken from the margin of an actively growing colony was inoculated on to the centre of each plate. Plates containing the same volume of methanol (without crude extract) served as negative controls and plates containing carbendazim (20 mg/ml) served as positive controls. Three independent biological replicates were performed, each with three technical replicates. The cultures were incubated at 28 °C in the dark for 3–5 days. When the negative control colonies reached approximately two-thirds of the plate area, the colony diameter was measured using the cross-plot method and the mycelial growth inhibition rate was calculated according to the formula:

$$\text{ Inhibition rate (\textbackslash{}\%) }=\frac{D_{1}-D_{2}}{D_{1}}\times 100\%$$

where D_1_ is the colony diameter (mm) of the negative control and D_2_ is the colony diameter (mm) of the treatment group.

Antibacterial activity was assessed against *Staphylococcus aureus* and *Escherichia coli* using the paper disc diffusion method. Bacteria-incorporated LA (Luria–Bertani agar) plates were prepared by inoculating molten LA (cooled to 45 °C) with a bacterial suspension to obtain a final concentration of 1 × 10^6^ CFU/ml in the medium. Each disc was loaded with 20 μl of the crude extract solution (20 mg/ml) by pipetting the solution directly on to the disc. Streptomycin solutions at 20 µg/ml and 100 µg/ml were used as positive controls and methanol-loaded discs of the same size served as negative controls. The plates were incubated at 37 °C for 24 h, after which the inhibition zone diameters were measured. Three independent biological replicates were performed, each with three technical replicates.

## Results

### Phylogenetic analyses

**Figure 1.**
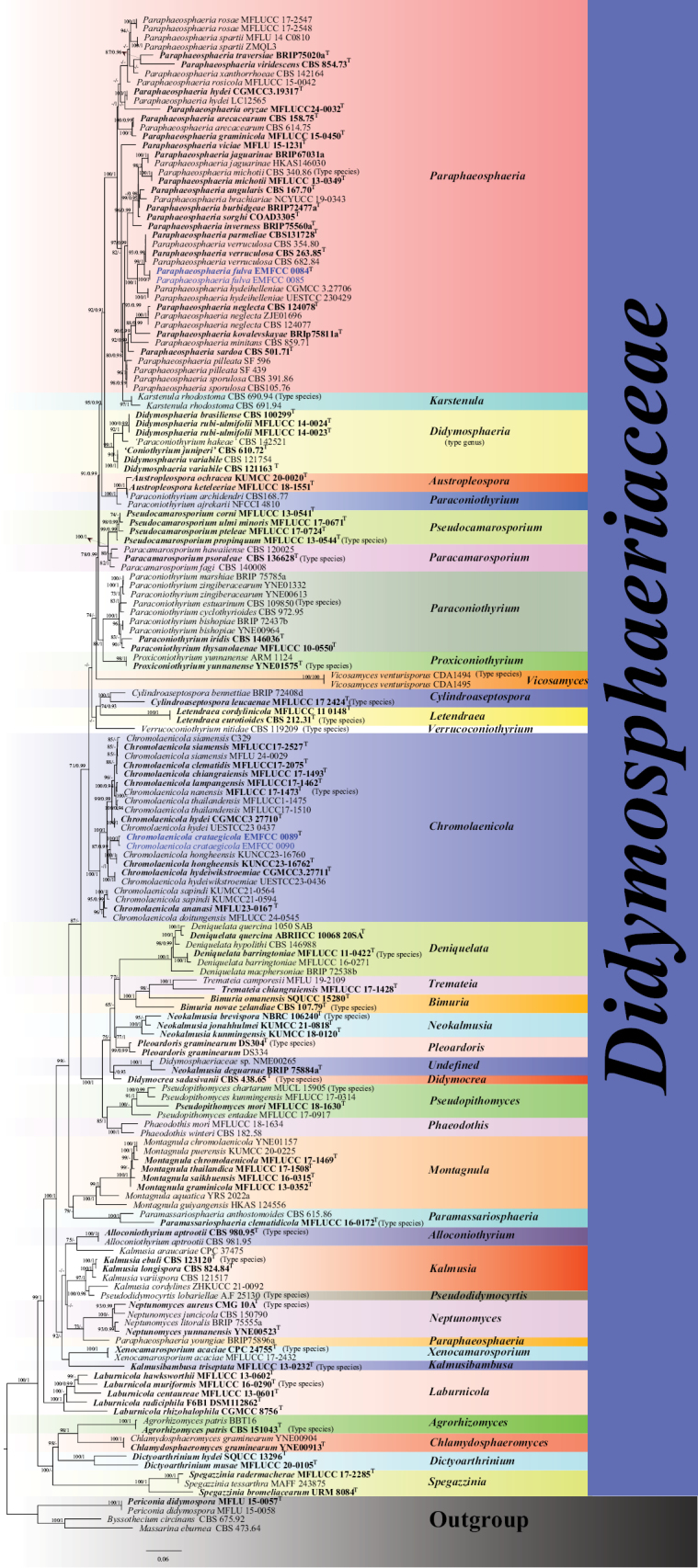
Phylogram of *Didymosphaeriaceae*, based on ITS, LSU, SSU, *rpb*2 and *tef*1-α genes. Maximum Likelihood bootstrap support values ≥ 70% (ML) and Bayesian posterior probabilities ≥ 0.90 (BYPP) are given at the nodes as ML/BYPP. The tree is rooted with *Periconia didymospora* (MFLU 15-0057, MFLU 15-0058), *Byssothecium circinas* CBS 675.92 and *Massarina eburnea* CBS 473.64. The new isolates are in bold blue. The type-derived strains are indicated in bold and marked with superscript capital ^T^.

Multi-locus phylogenetic analysis was conducted on the ITS, LSU, SSU, *tef*1-*α* and *rpb*2 sequence data of 169 *Didymosphaeriaceae* strains, including four newly-sequenced strains and the remaining 165 strains obtained through BLAST search (NCBI) and recent literature (Mapook et al. 2020; Phukhamsakda et al. 2020; Htet et al. 2024; Afshari et al. 2025; Chen et al. 2025; Du et al. 2025; Wanasinghe et al. 2025). *Periconia didymospora* (MFLU 15-0057 and MFLU 15-0058), *Byssothecium circinas* CBS 675.92 and *Massarina eburnea* CBS 473.64 were selected as the outgroup (Chen et al. 2025; Wanasinghe et al. 2025). The best-scoring RAxML tree with a final likelihood value of -45472.976090 was presented (Fig. 1). The matrix had 2535 distinct alignment patterns, with 52.62% of undetermined characters or gaps. Estimated base frequencies were as follows: A = 0.239229, C = 0.250921, G = 0.271693, T = 0.238157; substitution rates: AC = 1.467131, AG = 3.053274, AT = 1.615580, CG = 1.118994, CT = 7.100056, GT = 1.000000; gamma distribution shape parameter *α* = 0.430588. In the BYPP analysis, 13601 trees were sampled after the 20% burn-in with a stop value of 0.009994.

In ITS gene analysis, different genera within the *Didymosphaeriaceae* were clearly distinguishable, but differentiation between species within the same genus was not achievable (Suppl. material 1: fig. S1). This observation is consistent with previous reports that ITS exhibits high interspecific variation, but relatively low intraspecific variation at the genus level (Schoch et al. 2012). In contrast, the SSU and LSU genes showed ambiguous resolution even at the genus level (Suppl. material 1: figs S2, S3), consistent with their known conservation across a broad range of fungal taxa (James et al. 2006). Therefore, species identification within *Didymosphaeriaceae* requires a multi-locus phylogenetic approach utilising ITS, SSU, LSU, *tef*1-*α* and *rpb*2. In multi-locus phylogenetic analysis, the classification within *Didymosphaeriaceae* was clearly delineated, with each species also precisely defined. Our strains *Chromolaenicola crataegicola* (EMFCC 0089 and EMFCC 0090), formed a sister subclade with *C. hongheensis* (KUNCC23 16760 and KUNCC23 16762), with moderate support (87% ML/0.99 BYPP). *Paraphaeosphaeria fulva* (EMFCC 0084 and EMFCC 0085) clustered with *P. verruculosa* (CBS 682.84 and CBS 263.85) and *P. parmeliae* (CBS 131728T) with high support values (99% ML/1 BYPP) (Fig. 1).

### Taxonomy

#### Chromolaenicola crataegicola

Taxon classificationFungiPleosporalesDidymosphaeriaceae

W.X. Su, B. Zhang & X. Li sp. nov.

A8C874A4-FF73-5A78-8EF7-847FAAE39569

863302

Fig. 2

##### Etymology.

Refers to the host genus *Crataegus*.

**Figure 2.**
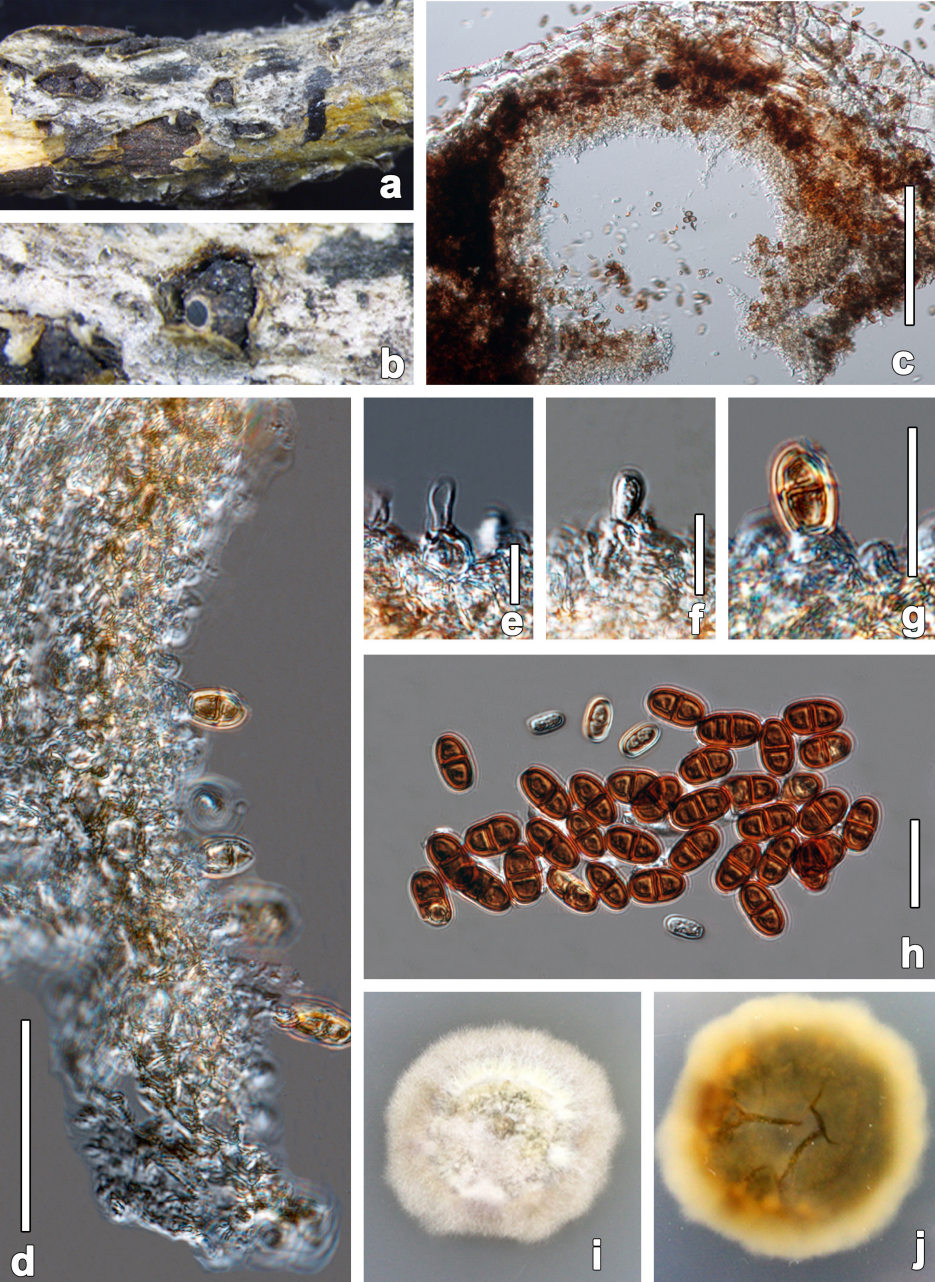
*Chromolaenicola crataegicola* (HMJAU 64871, holotype). **a, b**. Appearance of conidiomata on host substrate; **c**. Vertical section of partial conidiomata; **d**. Section of partial conidioma wall; **e, g**. Conidiogenous cells and conidia; **h**. Conidia; **i, j**. Culture characteristics on PDA. Scale bars: 100 µm (**c**); 10 µm (**d**); 5 µm (**e**); 10 µm (**f, h**).

##### Holotype.

HMJAU 64871.

##### Description.

Saprobic on dead twigs of *Crataegus*.

***Sexual morph***: Undetermined. ***Asexual morph***: Coelomycetous. *Conidiomata* pycnidial, 220–413 µm × 290–427 µm, solitary, immersed, glabrous, gregarious black bulges on host surface, spherical to subspherical, coriaceous, black. *Pycnidial* wall 17–32 µm wide, thin-walled, composed of cells of *textura angularis*, dark brown on the outside to gradually becoming lighter towards the inside, inner layer subhyaline. *Conidiophores* reduced to conidiogenous cells. *Conidiogenous* cells oblate or oblong or inconspicuous, hyaline. *Conidia* 6–9 µm × 3–5 µm (x̄ = 7 µm × 3.9 µm, n = 50), oblong or oval, initially hyaline to yellow without septa, dark reddish-brown at maturity with 1-septum, part of the spores slightly constricted in the middle, internal cellular depression, smooth-walled.

##### Cultural characteristics.

Colonies on PDA, round, 36.1–40.6 mm in diameter, with a central elevation and irregular margins, white, rugose, dense; the reverse side transitions from yellow to white from the centre to the periphery, exhibiting fissures.

##### Material examined.

China • Jilin Province, Changchun, from the branches of *Crataegus* sp., 15 March 2021, Wenxin Su, SWX8.1 (HMJAU 64871, ***holotype***); ex-type EMFCC 0089. • *Ibid*., isotype HMJAU 64894, ex-isotype EMFCC 0090.

##### GenBank accession numbers.

EMFCC 0089: ITS = PZ096738; LSU = PZ096746; SSU = PZ096742; *rpb*2 = PZ213744; *tef*1-*α* = PZ213748; EMFCC 0090: ITS = PZ096739; LSU = PZ096747; SSU = PZ096743; *rpb*2 = PZ213745; *tef*1-*α* = PZ213749.

##### Notes.

Multi-locus phylogenetic analysis showed that *Chromolaenicola crataegicola* was sister to *C. hongheensis* (KUNCC23 16760 and KUNCC23 16762) (ML/BP = 81%/1.00) and formed a distinct lineage. There were 1.45% (8/553 bases), 4.81% (14/291), 1.76% (15/853) base differences in the ITS, *rpb*2 and *tef*1-*α* genes between *C. crataegicola* (EMFCC 0089) and *C. hongheensis* (KUNCC23-16762), excluding gaps. Morphologically, the two species are clearly distinguished. The conidiomata of *C. crataegicola* are notably larger than those of *C. hongheensis* (220–413 µm × 290–427 μm vs. 150–200 µm × 120–250 μm) (Wanasinghe et al. 2025). *C. hongheensis* exhibits more pronounced conidiogenous cells compared to *C. crataegicola*. The conidia of *C. crataegicola* is dark reddish-brown after maturity with 1-septum, part of the spores slightly constricted in the septum and internal cellular depression, whereas those of *C. hongheensis* become brown after maturity, with 1-septum, guttulate, rough-walled and verruculose at maturity (Wanasinghe et al. 2025). Therefore, based on morphological characteristics and phylogenetic analysis, we introduce *C. crataegicola* as a new species in *Chromolaenicola*.

#### Paraphaeosphaeria fulva

Taxon classificationFungiPleosporalesDidymosphaeriaceae

W.X. Su, B. Zhang & X. Li sp. nov.

DB1374B3-0D7A-5215-A839-D9A3C1137CCA

863303

Fig. 3

##### Etymology.

Refers to pale yellow mature conidia.

**Figure 3.**
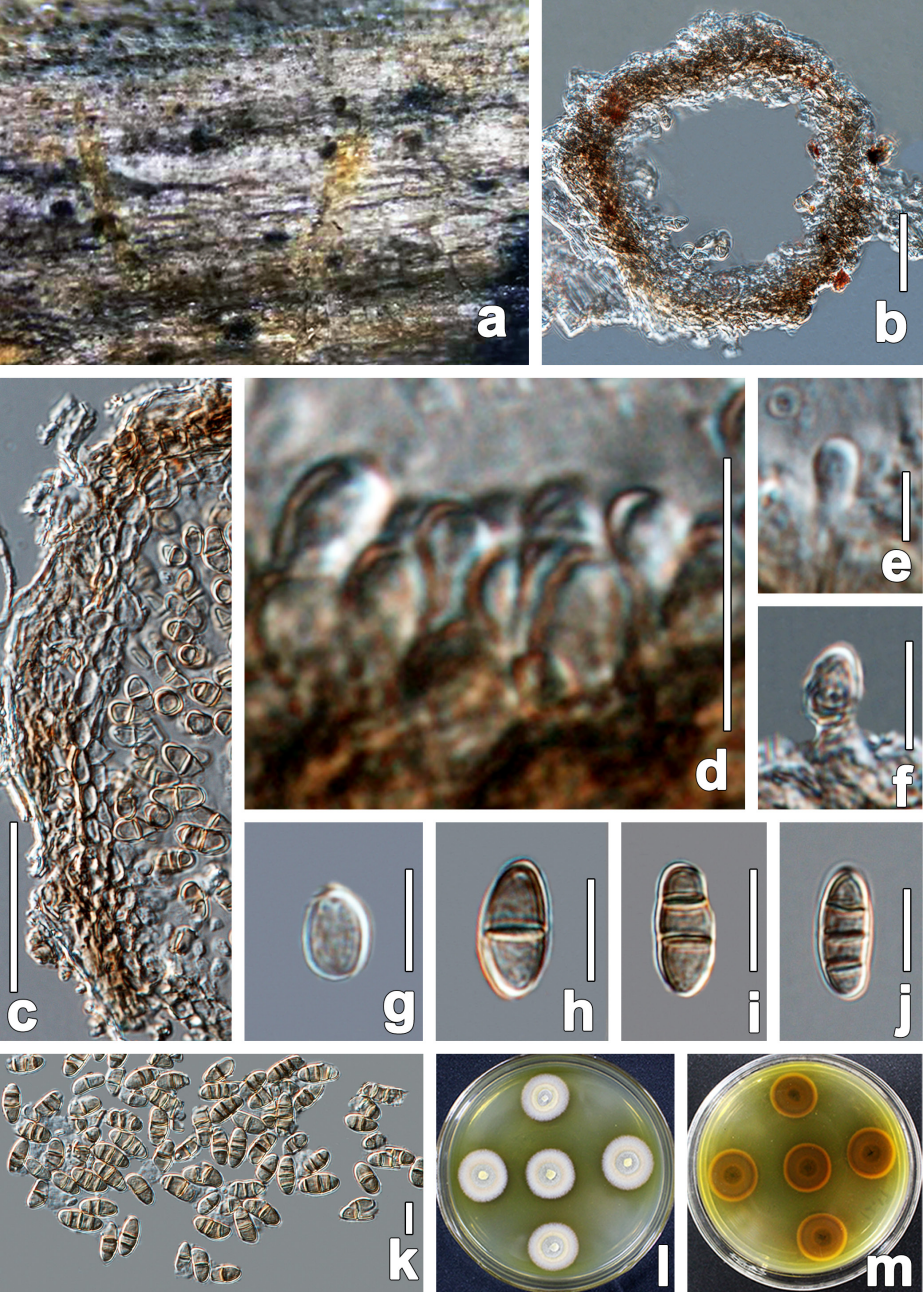
*Paraphaeosphaeria fulva* (HMJAU 70082, holotype) **a**. Appearance of conidiomata on host substrate; **b**. Vertical section of partial conidiomata; **c**. Section of partial conidioma wall; **d, f**. Conidiogenous cells and conidia; **g–k**. Conidia; **l, m**. Culture characteristics on PDA. Scale bars: 20 µm (**b, c**); 5 µm (**d, f–k**); 2 µm (**e**).

##### Holotype.

HMJAU 70082.

##### Description.

Saprobic on an unknown dead branch.

***Sexual morph***: Undetermined. ***Asexual morph***: Coelomycetous. *Conidiomata* pycnidial, 72–86 µm × 74–122 µm (x̄ = 80 µm × 93 µm), buried under the epidermis of the host, black spots that are raised, subglobose, scattered and solitary. *Pycnidial wall* 7–15 µm, composed of 4–7 layers of *textura angularis*, thin, brown on the outer side, yellow on the inner side and transparent cells in the inner layer. *Conidiophores* reduced to conidiogenous cells. *Conidiogenous* cells hyaline, enteroblastic, oblate or ampullate, lining the conidiomata cavity. *Conidia* 5.4–9.6 µm × 2.6–4.2 µm (x = 7.1 µm × 3.4 µm), fusiform or reniform, 1–3 septa, initially hyaline and eventually turning pale yellow, partially curved in the middle, with smooth walls.

##### Cultural characteristic.

Single conidium germinated on PDA, dark, 25 °C for 24 h. After two weeks, the colony diameter of 18.2–19.6 mm, with round shapes, regular margins, dense growth and greyish-white. Colonies displayed distinct concentric circles and pale yellow concentric rings, producing yellow pigmentation on the culture medium; reverse side appeared yellowish-brown with white margins and clearly defined concentric circles.

##### Material examined.

China • Jilin Province, Changchun, from the branches of dead wood, 15 March 2021, Wenxin Su, SWX21 (HMJAU 70082, ***holotype***); ex-type EMFCC 0084. • *Ibid*., isotype HMJAU 70083, ex-isotype EMFCC 0085.

##### GenBank accession numbers.

EMFCC 0084: ITS = PZ096740; LSU = PZ096748; SSU = PZ096744; *tef*1-*α* = PZ213746; EMFCC 0085: ITS = PZ096741; LSU = PZ096749; SSU = PZ096745; *tef*1-*α* = PZ213747.

##### Notes.

Multi-locus phylogeny analysis showed that two new strains (EMFCC 0084 and EMFCC 0085) clustered with *Paraphaeosphaeria verruculosa* (CBS 263.85, CBS 682.84, CBS 354.80) and *P. parmeliae* (CBS 131728) with high support (99% ML/1 BYPP; Fig. 1). Morphologically, our new collection has distinct differences from its close relatives *P. verruculosa* (CBS 263.85) and *P. parmeliae* (CBS 131728). The conidiogenous cells of *P. verruculosa* are discrete, globose to broadly ampulliform, phialidic, 4–6 µm × 3–4 µm (Verkley et al. 2014), whereas those of *P. fulva* are hyaline, oblate or ampullate, lining the conidiomatal cavity and those of *P. parmeliae* are brown and ampulliform, 4.5–6.5 µm × 3.5–7 µm (Thippawan et al. 2014). The conidia of *P. verruculosa* are globose, subglobose to ellipsoid, initially hyaline with 3–7 small oil droplets; the mature conidial wall is orange-brown and verruculose, aseptate, 3–6 µm × 2.5–5 µm (Verkley et al. 2014). In contrast, the conidia of *P. fulva* are fusiform or reniform, initially hyaline and eventually turning pale yellow, partially curved in the middle, with 1–3 septa, 5.4–9.6 µm × 2.6–4.2 µm, whereas those of *P. parmeliae* are globose, brown, aseptate, thick-walled and smooth to rough, 3–4.5 µm × 3–4.5 µm (Thippawan et al. 2014). Thus, based on phylogenetic evidence and morphological characteristics, the new isolates are identified as *P. fulva*.

### Antibacterial and antifungal activities of crude extracts

**Figure 4.**
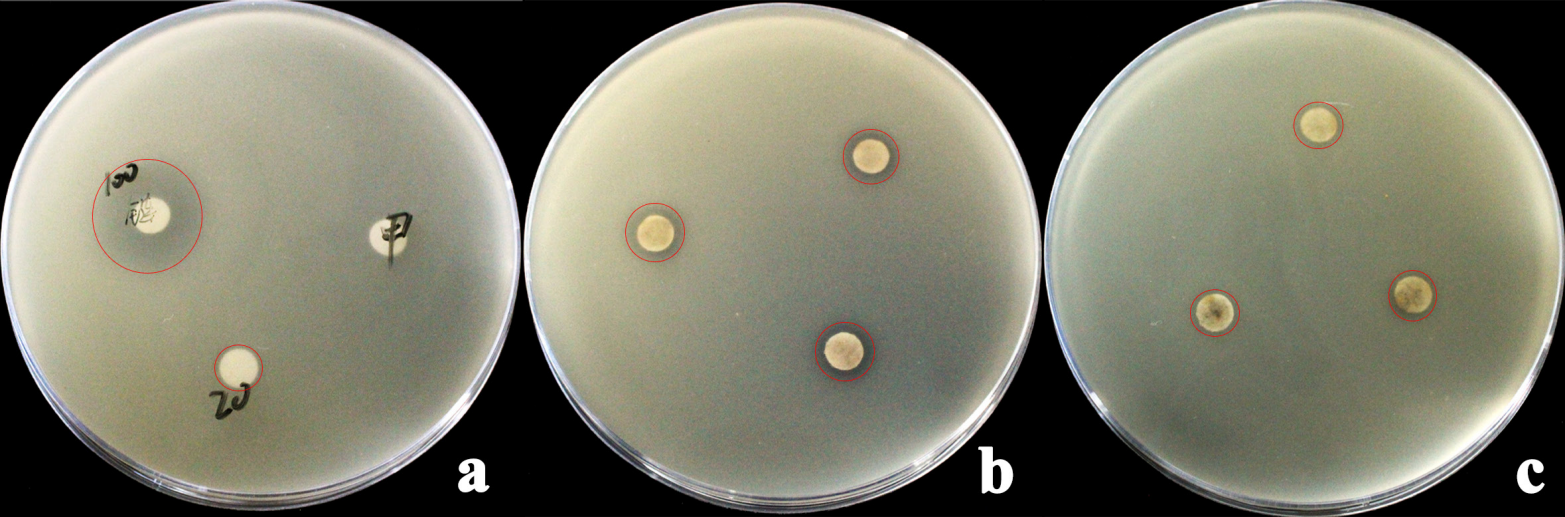
Results of bacterial inhibition by the secondary metabolites ethyl acetate extract. **a**. Contrast; **b**. *Chromolaenicola crataegicola*; **c**. *Paraphaeosphaeria fulva*.

Antibacterial test results indicated that the crude extracts from both strains inhibited *Staphylococcus aureus* (Fig. 4), but showed no activity against *Escherichia coli*. The crude extract of *C. crataegicola* produced an inhibition zone of 8.38 ± 0.36 mm against *S. aureus*, which was significantly larger than that of *P. fulva* (7.09 ± 0.29 mm) (*p* < 0.05) (Table 2). The positive control (streptomycin, 20 µg/ml) produced an inhibition zone of 7.26 mm (Fig. 4). Although the inhibition zones produced by the crude extracts were similar to that of the positive control, the crude extracts were tested at a much higher concentration (20 mg/ml vs. 20 µg/ml). Therefore, the antibacterial activity of the crude extracts was modest compared with the pure antibiotic at equivalent concentrations. Between the two stains, *C. crataegicola* showed slightly stronger inhibitory activity against *S. aureus* than *P. fulva*.

**Table 2.** The inhibition zone diameters of *C. crataegicola* and *P. fulva*.

**Treatment**	**Concentration**	**Inhibition zone (mm)**
*C. crataegicola*	20 mg/ml	8.38 ± 0.36 a
*P. fulva*	20 mg/ml	7.09 ± 0.29 b
Streptomycin	20 µg/ml	7.26
Methanol	—	0

Different letters within the same column indicate significant differences at *p* < 0.05.

**Figure 5.**
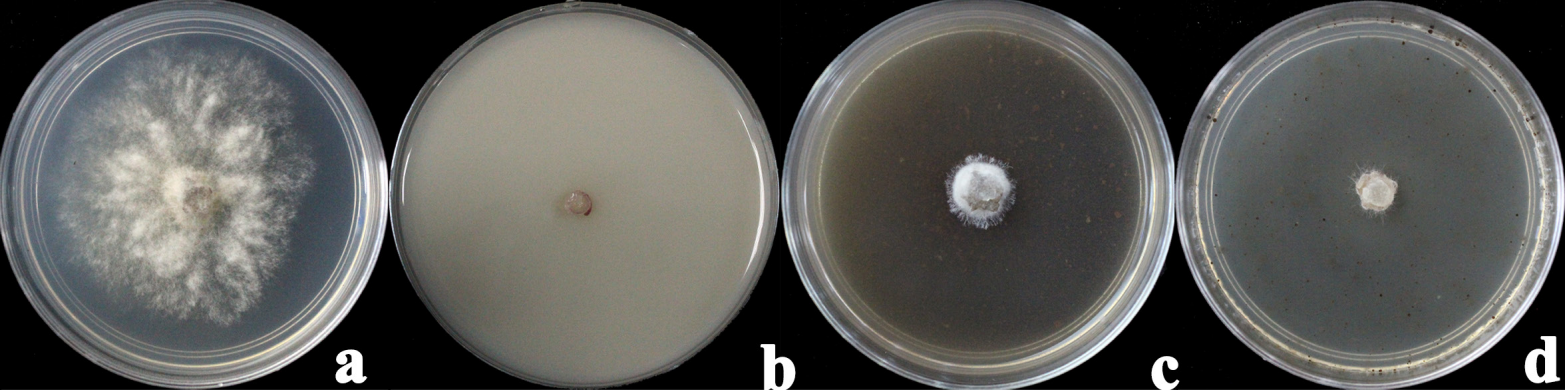
Results of Inhibition of *Botrytis cinerea* by the secondary metabolites ethyl acetate extract. **a**. Contrast; **b**. Carbendazim; **c**. *Chromolaenicola crataegicola*; **d**. *Paraphaeosphaeria fulva*.

**Figure 6.**
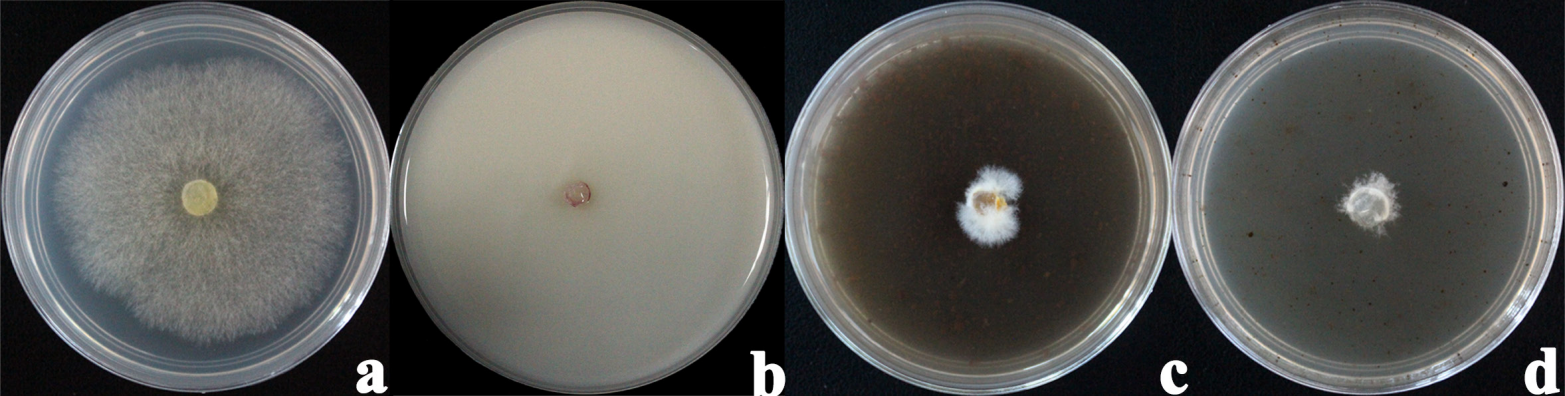
Results of Inhibition of *Cladobotryum mycophilum* by the secondary metabolites ethyl acetate extract. **a**. Contrast; **b**. Carbendazim; **c**. *Chromolaenicola crataegicola*; **d**. *Paraphaeosphaeria fulva*.

**Figure 7.**
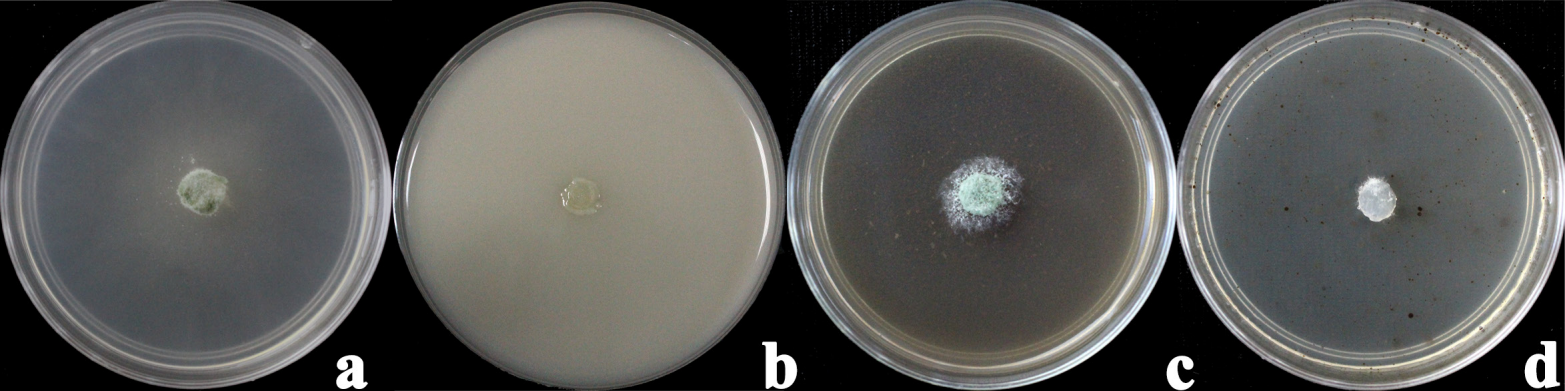
Results of Inhibition of *Trichoderma harzianum* by the secondary metabolites ethyl acetate extract. **a**. Contrast; **b**. Carbendazim; **c**. *Chromolaenicola crataegicola*; **d**. *Paraphaeosphaeria fulva*.

The crude extract of *P. fulva* exhibited significantly stronger antifungal activity than that of *C. crataegicola* against *B. cinerea* (85.22 ± 2.99% vs. 81.00 ± 1.49%, *p* < 0.05) and *T. harzianum* (88.86 ± 1.14% vs. 80.63 ± 2.65%, *p* < 0.05). No significant difference was observed against *Cl. mycophilum* (76.22 ± 5.49% vs. 77.13 ± 2.58%, *p* = 0.951) (Table 3). The positive control (carbendazim, 20 mg/ml) achieved 100% inhibition against all three fungi (Figs 5, 6, 7). These results showed that both crude extracts possess moderate antifungal activity, with *P. fulva* showing relatively stronger inhibition than *C. crataegicola*.

**Table 3.** Inhibition rates (%) of crude extracts from *C. crataegicola* and *P. fulva* against *B. cinerea*, *Cl. Mycophilum* and *T. harzianum*.

**Treatment**	***Cl. mycophilum***	***B. cinerea***	***T. harzianum***
*C. crataegicola*	77.13 ± 2.58	81.00 ± 1.49 b	80.63 ± 2.65 b
*P. fulva*	76.22 ± 5.49	85.22 ± 2.99 a	88.86 ± 1.14 a

Different letters within the same column indicate significant differences at *p* < 0.05.

## Discussion

In this study, we introduced two novel species, *Chromolaenicola crataegicola* and *Paraphaeosphaeria fulva*, to family *Didymosphaeriaceae* and preliminarily evaluated the antimicrobial activity of their crude extracts using the mycelial growth rate and inhibition zone methods. Both novel species inhibited *Staphylococcus aureus*, *Botrytis cinerea*, *Cladobotryum mycophilum* and *Trichoderma harzianum*, but showed no significant activity against *Escherichia coli*.

Our multi-locus phylogeny is largely congruent with the framework of Chen et al. (2025). Consistent with their study, ‘*Paraconiothyrium hakeae’* CBS 142521 and ‘*Coniothyrium juniperi’* CBS 610.72 grouped with *Didymosphaeria*, whereas *‘Neokalmusia deguarnae’* BRIP 75884a clustered with *Didymosphaeriaceae* sp. NME00265 formed a distinct clade (Fig. 1), representing an undescribed genus. This confirms that several misidentified strains require re-classification and highlights the necessity of multi-locus phylogenetic analyses combined with morphological characteristics for accurate generic delimitation.

Studies on *Chromolaenicola* have primarily focused on taxonomy and phylogenetic analyses, with limited attention to bioactivity. Mapook et al. (2020) conducted antimicrobial pre-screening of four *Chromolaenicola* species: *C. nanensis*, *C. chiangraiensis*, *C. lampangensis* and *C. thailandensis*. None of these species inhibited *Escherichia coli* or *Bacillus subtilis* and only *C. nanensis* and *C. lampangensis* showed weak activity against *Mucor plumbeus*. In contrast, our study shows that *C. crataegicola* exhibits inhibitory activity against *S. aureus* and three fungi, suggesting previously unrecognised antimicrobial potential within this genus.

Due to the coniothyrium-like asexual morphology of *Paraphaeosphaeria*, some species have been misclassified into other genera. Verkley et al. (2014) established *Paraconiothyrium* for asexual morphs associated with *Paraphaeosphaeria*, including *Coniothyrium sporulosum* and *Co. minitans*. However, subsequent multi-locus phylogenetic analyses recombined these two species back into *Paraphaeosphaeria* as *P. minitans* and *P. sporulosa* (Verkley et al. 2014). Similarly, the isolate of *Paraphaeosphaeria parmeliae* was originally identified as *Phoma foliaceiphila* (Thippawan et al. 2014). Thus, multi-locus phylogenetic analysis for accurate species identification in *Paraphaeosphaeria* is important.

*Paraphaeosphaeria* species are recognised as promising sources of bioactive secondary metabolites (Shao 2015; Hu et al. 2020; Sun et al. 2020), with antifungal activity reported from *P. verruculosa* and *P. sporulosa* (Hu et al. 2020; Chen et al. 2022). This study shows that the crude extract of *P. fulva* exhibited significant antifungal activity against all three tested phytopathogenic fungi, with notably stronger inhibition against *T. harzianum* (88.86%) and *B. cinerea* (85.22%) than *C. crataegicola* (p < 0.05). These findings are consistent with previous reports highlighting the antifungal potential of *Paraphaeosphaeria* species. Our study further confirms that the genus *Paraphaeosphaeria* represents a promising source of antifungal natural products, with *P. fulva* showing greater activity than *C. crataegicola* within the same family. Further investigation into the isolation and characterisation of bioactive compounds from *P. fulva* is warranted.

However, the crude extracts tested are complex mixtures and the observed activity cannot be attributed to specific compounds. Additionally, the concentration used for the antibacterial activity test (20 mg/ml) was significantly higher than that of the positive control. Our findings suggest that *Chromolaenicola* and *Paraphaeosphaeria* represent underexplored sources for natural product discovery, with potential applications in biological control. Further studies are required to identify the active compounds responsible for the observed bioactivity and to evaluate their antibacterial and antifungal properties.

## Supplementary Material

XML Treatment for Chromolaenicola crataegicola

XML Treatment for Paraphaeosphaeria fulva

## Additional information

### Artificial Intelligence (AI) use

The authors accept full responsibility for the content of the manuscript, including the disclosure of any use of AI.

No AI tools were used in the preparation of this manuscript.

### Author ORCIDs

W.-X. Su https://orcid.org/0000-0002-5470-5853

R. Tieliwadi https://orcid.org/0009-0007-1322-3296

B. Zhang https://orcid.org/0000-0001-9508-8188

X.-H. Yuan https://orcid.org/0000-0003-0661-5332

X. Li https://orcid.org/0000-0002-1230-6467

### Data availability

All sequences generated in this study were submitted to GenBank and all the data from this study are available in the main text or Supplementary information.

## Supplementary materials

Supplementary material 1 Supplementary images Data type docx https://binary.pensoft.net/file/1720209This dataset is made available under the Open Database License (http://opendatacommons.org/licenses/odbl/1.0/). The Open Database License (ODbL) is a license agreement intended to allow users to freely share, modify, and use this Dataset while maintaining this same freedom for others, provided that the original source and author(s) are credited. Wen-Xin Su, Ranagul Tieliwadi, Bo Zhang, Xiao-Hui Yuan, Xiao Li

## References

[B1] Afshari N, Noorabadi MT, McKenzie EHC, Pumas C, Bhunjun CS, Jayawardena RS, Gomes de Farias AR, Phukhamsakda C, Jeewon R, Chaharmiri-Dokhaharani S, Suwannarach N, Kumla J, Al-Otibi F, Hyde KD, Lumyong S (2025) Taxonomy and diversity of woody litter microfungi associated with six phylogenetically related host species in Doi Tung national park, Chiang Rai, Thailand. Mycosphere 16(1): 4783. 10.5943/mycosphere/16/1/36

[B2] Aptroot A (1995) A monograph of *Didymosphaeria*. Studies in Mycology 37: 1–160.

[B3] Ariyawansa HA, Tanaka K, Thambugala KM, Phookamsak R, Tian Q, Camporesi E, Hongsanan S, Monkai J, Wanasinghe DN, Mapook A, Chukeatirote E, Kang JC, Xu JC, McKenzie EHC, Jones EBG, Hyde KD (2014) A molecular phylogenetic reappraisal of the *Didymosphaeriaceae* (= *Montagnulaceae*). Fungal Diversity 68: 69–104. 10.1007/s13225-014-0305-6

[B4] Chen KY, Mao LJ, Li KL, Zhang CL (2025) Exploring the dominant endophytic pleosporalean fungi in *Poaceae* plants: taxonomic novelties within the suborder *Massarineae*. Mycology 17(2): 481–508. 10.1080/21501203.2025.2569933 PMC13267030 42305148

[B5] Chen Q, Yu JJ, He J, Feng T, Liu JK (2022) Isobenzofuranones and isocoumarins from kiwi endophytic fungus *Paraphaeosphaeria sporulosa* and their antibacterial activity against *Pseudomonas syringae* pv. *actinidiae*. Phytochemistry 195: 113050. 10.1016/j.phytochem.2021.113050 34906836

[B6] Cui LY, Ma ZN, Wang DF, Niu YB (2022) Ultrasound-assisted extraction, optimization, isolation, and antioxidant activity analysis of flavonoids from *Astragalus membranaceus* stems and leaves. Ultrasonics Sonochemistry 90: 106190. 10.1016/j.ultsonch.2022.106190 PMC9554832 36215890

[B7] Darriba D, Taboada GL, Doallo R, Posada D (2012) jModelTest 2: more models, new heuristics and parallel computing. Nature Methods 9(8): 772. 10.1038/nmeth.2109 PMC4594756 22847109

[B8] Dissanayake LS, Wijayawardene NN, Samarakoon MC, Hyde KD, Kang JC (2021) The taxonomy and phylogeny of *Austropleospora ochracea* sp. nov. (*Didymosphaeriaceae*) from Guizhou, China. Phytotaxa 491(3): 217–229. 10.11646/phytotaxa.491.3.2

[B9] Du HZ, Chi MF, Wu N, Dissanayake AJ, Liu NG, Cheewangkoon R, Liu JK (2025) Taxonomic and phylogenetic insights to *Dothideomycetes* and *Sordariomycetes* associated with medicinal plants in Southwestern China. Mycosphere 16(2): 179–343. 10.5943/mycosphere/16/2/2

[B10] Eriksson O (1967) On graminicolous pyrenomycetes from Fennoscandia. 2. *Phragmosporous* and *scolecosporous* species. Arkiv för Botanik.

[B11] Gao H, Wang YN, Luo Q, Yang LY, He XX, Wu J, Konthorn K, Pongthep W, Zhu WM, Wang Y (2021) Bioactive metabolites from acid-tolerant fungi in a thai mangrove sediment. Frontiers in Microbiology 11: 609952. 10.3389/FMICB.2020.609952 PMC7862741 33552019

[B12] Hongsanan S, Hyde KD, Phookamsak R, Wanasinghe DN, McKenzie EHC, Sarma VV, Boonmee S, Lücking R, Bhat J, Liu NG (2020) Refined families of *Dothideomycetes*: *Dothideomycetidae* and *Pleosporomycetidae*. Mycosphere 11(1): 1553–2107. 10.5943/mycosphere/11/1/13

[B13] Htet ZH, Mapook A, Chethana KWT (2024) Molecular taxonomy reveals new records of *Chromolaenicola* (*Didymosphaeriaceae*, *Pleosporales*) and potential antibacterial properties. Studies in Fungi 9: e006. 10.48130/sif-0024-0006

[B14] Hu M, Yang XQ, Wang CF, Zhao TD, Wang DL, Yang YB, Ding ZT (2020) Paraverrucsins A-F, Antifeedant, and Antiphytopathogenic Polyketides from Rhizospheric *Paraphaeosphaeria verruculosa* and Induced Bioactivity Enhancement by Coculturing with Host Plant *Dendrobium officinale*. ACS Omega 5(47): 30596–30602. 10.1021/acsomega.0C04548 PMC7711696 33283108

[B15] Hyde KD, Noorabadi MT, Thiyagaraja V, He MQ, Johnston PR, Wijesinghe SN, Armand A, Biketova AY, Chethana KWT, Erdoğdu M, Ge ZW, Groenewald JZ, Hongsanan S, Kušan I, Leontyev DV, Li DW, Lin CG, Liu NG, Maharachchikumbura SSN, Matočec N, May TW, McKenzie EHC, Mešić A, Perera RH, Phukhamsakda C, Piątek M, Samarakoon MC, Selcuk F, Senanayake IC, Tanney JB, Tian Q, Vizzini A, Wanasinghe DN, Wannasawang N, Wijayawardene NN, Zhao RL, Zvyagina E (2024) The 2024 outline of *Fungi* and fungus-like taxa. Mycosphere 15(1): 5146–6239. 10.5943/mycosphere/15/1/25

[B16] James TY, Kauff F, Schoch CL, Matheny PB, Hofstetter V, Cox CJ, Celio G, Gueidan C, Fraker E, Miadlikowska J, Lumbsch HT, Rauhut A, Reeb V, Arnold AE, Amtoft A, Stajich JE, Hosaka K, Sung GH, Johnson D, O’Rourke B, Crockett M, Binder M, Curtis JM, Slot JC, Wang Z, Wilson AW, Schüßler A, Longcore JE, O’Donnell K, Mozley-Standridge S, Porter D, Letcher PM, Powell MJ, Taylor JW, White MM, Griffith GW, Davies DR, Humber RA, Morton JB, Sugiyama J, Rossman AY, Rogers JD, Pfister DH, Hewitt D, Hansen K, Hambleton S, Shoemaker RA, Kohlmeyer J, Volkmann-Kohlmeyer B, Spotts RA, Serdani M, Crous PW, Hughes KW, Matsuura K, Langer E, Langer G, Untereiner WA, Lücking R, Büdel B, Geiser DM, Aptroot A, Diederich P, Schmitt I, Schultz M, Yahr R, Hibbett DS, Lutzoni F, McLaughlin DJ, Spatafora JW, Vilgalys R (2006) Reconstructing the early evolution of *Fungi* using a six-gene phylogeny. Nature 443(7113): 818–822. 10.1038/nature05110 17051209

[B17] Jayasiri SC, Hyde KD, Jones EBG, McKenzie EHC, Jeewon R, Phillips AJL, Bhat DJ, Wanasinghe DN, Liu JK, Lu YZ, Kang JC, Xu J, Karunarathna SC (2019) Diversity, morphology and molecular phylogeny of *Dothideomycetes* on decaying wild seed pods and fruits. Mycosphere 10(1): 1–186. 10.5943/mycosphere/10/1/1

[B18] Katoh K, Rozewicki J, Yamada KD (2019) MAFFT online service: multiple sequence alignment, interactive sequence choice and visualization. Briefings in Bioinformatics 20(4): 1160–1166. 10.1093/bib/bbx108 PMC6781576 28968734

[B19] Khodaei S, Arzanlou M, Babai-Ahari A, Rota-Stabelli O, Pertot I (2019) Phylogeny and evolution of *Didymosphaeriaceae (Pleosporales)*: new Iranian samples and hosts, first divergence estimates, and multiple evidences of species mis-identifications. Phytotaxa 424(3): 131–146. 10.11646/phytotaxa.424.3.1

[B20] Larsson A (2014) AliView: A fast and lightweight alignment viewer and editor for large datasets. Bioinformatics 30: 3276–3278. 10.1093/bioinformatics/btu531 PMC4221126 25095880

[B21] Maharachchikumbura S, Wanasinghe DN, Cheewangkoon R, Abdullah M, Al-Sadi (2021) Uncovering the hidden taxonomic diversity of fungi in Oman. Fungal Diversity 106(1): 1–40. 10.1007/S13225-020-00467-1

[B22] Malarvizhi K, Murali TS, Kumaresan V (2022) First report of *Paraphaeosphaeria angularis* as endophyte in sugarcane (*Saccharum officinarum*) from India. The Indian Journal of Agricultural Sciences 92(12): 1511–1513. 10.56093/ijas.v92i12.128491

[B23] Mapook A, Hyde KD, Mckenzie E, Jones EBG, Bhat DJ, Jeewon R, Stadler M, Samarakoon MC, Malaithong M, Tanunchai B, Buscot F, Wubet T, Purahong W (2020) Taxonomic and phylogenetic contributions to fungi associated with the invasive weed *Chromolaena odorata* (Siam weed). Fungal Diversity 101: 1–175. 10.1007/s13225-020-00444-8

[B24] Phookamsak R, Hyde KD, Jeewon R, Bhat DJ, Jones EBG, Maharachchikumbura SSN, Raspé O, Karunarathna SC, Wanasinghe DN, Hongsanan S, Doilom M, Tennakoon DS, Machado AR, Firmino AL, Ghosh A, Karunarathna A, Mešić A, Dutta AK, Thongbai B, Devadatha B, Norphanphoun C, Senwanna C, Wei D, Pem D, Ackah FK, Wang GN, Jiang HB, Madrid H, Lee HB, Goonasekara ID, Manawasinghe IS, Kušan I, Cano J, Gené J, Li J, Das K, Acharya K, Raj KNA, Latha KPD, Chethana KWT, He MQ, Dueñas M, Jadan M, Martín MP, Samarakoon MC, Dayarathne MC, Raza M, Park MS, Telleria MT, Chaiwan N, Matočec N, de Silva NI, Pereira OL, Singh PN, Manimohan P, Uniyal P, Shang QJ, Bhatt RP, Perera RH, Alvarenga RLM, Nogal-Prata S, Singh SK, Vadthanarat S, Oh SY, Huang SK, Rana S, Konta S, Paloi S, Jayasiri SC, Jeon SJ, Mehmood T, Gibertoni TB, Nguyen TTT, Singh U, Thiyagaraja V, Sarma VV, Dong W, Yu XD, Lu YZ, Lim YW, Chen Y, Tkalčec Z, Zhang ZF, Luo ZL, Daranagama DA, Thambugala KM, Tibpromma S, Camporesi E, Bulgakov TS, Dissanayake AJ, Senanayake IC, Dai DQ, Tang LZ, Khan S, Zhang H, Promputtha I, Cai L, Chomnunti P, Zhao RL, Lumyong S, Boonmee S, Wen TC, Mortimer PE, Xu J (2019) Fungal diversity notes 929–1035: taxonomic and phylogenetic contributions on genera and species of fungi. Fungal Diversity 95: 1–273. 10.1007/s13225-019-00421-w

[B25] Phookamsak R, Hongsanan S, Bhat JD, Wanasinghe DN, Promputtha I, Suwannarach N, Kumla J, Xie N, Dawoud TM, Mortimer PE, Xu JC, Lumyong S (2024) Exploring ascomycete diversity in Yunnan II: Introducing three novel species in the suborder *Massarineae* (*Dothideomycetes*, *Pleosporales*) from fern and grasses. MycoKeys 104: 9–50. 10.3897/mycokeys.104.112149 PMC11040200 38665970

[B26] Phukhamsakda C, McKenzie EHC, Phillips AJL, Jones EBG, Bhat DJ, Stadler M, Bhunjun CS, Wanasinghe DN, Thongbai B, Camporesi E, Ertz D, Jayawardena RS, Perera RH, Ekanayake AH, Tibpromma S, Doilom M, Xu J, Hyde KD (2020) Microfungi associated with Clematis (*Ranunculaceae*) with an integrated approach to delimiting species boundaries. Fungal Diversity 102: 1–203. 10.1007/s13225-020-00448-4

[B27] Rambaut A (2018) FigTree v1.4.4. A graphical viewer of phylogenetic trees. University of Edinburgh, Edinburgh.

[B28] Rehner SA, Buckley E (2005) A Beauveria phylogeny inferred from nuclear ITS and EF1-*α* sequences: evidence for cryptic diversification and links to *Cordyceps* teleomorphs. Mycologia 97(1): 84–98. 10.3852/mycologia.97.1.84 16389960

[B29] Ren G, Wanasinghe DN, de Farias ARG, Hyde KD, Yasanthika E, Xu JC, Abhaya B, Thilini CKW, Gui H (2022) Taxonomic novelties of woody litter fungi (*Didymosphaeriaceae*, *Pleosporales*) from the Greater Mekong Subregion. Biology 11(11): 1660–1660. 10.3390/biology11111660 PMC9687740 36421373

[B30] Ronquist F, Huelsenbeck JP (2003) MrBayes 3: Bayesian phylogenetic inference under mixed models. Bioinformatics 19: 1572–1574. 10.1093/bioinformatics/btg180 12912839

[B31] Samarakoon BC, Wanasinghe DN, Samarakoon MC, Phookamsak R, McKenzie EHC, Chomnunti P, Hyde KD, Lumyong S, Karunarathna SC (2020) Multi-gene phylogenetic evidence suggests *Dictyoarthrinium* belongs in *Didymosphaeriaceae* (*Pleosporales*, *Dothideomycetes*) and *Dictyoarthrinium musae* sp. nov. on *Musa* from Thailand. MycoKeys 71: 101–118. 10.3897/mycokeys.71.55493 PMC7423779 32855605

[B32] Schoch CL, Seifert KA, Huhndorf S, Robert V, Spouge JL, Levesque CA, Chen W, Fungal Barcoding Consortium, Bolchacova E, Voigt K, Crous PW, Miller AN, Wingfield MJ, Aime MC, An K, Bai F, Barreto RW, Begerow D, Bergeron M, Blackwell M, Boekhout T, Bogale M, Boonyuen N, Burgaz AR, Buyck B, Cai L, Cai Q, Cardinali G, Chaverri P, Coppins BJ, Crespo A, Cubas P, Cummings C, Damm U, de Beer ZW, de Hoog GS, Del-Prado R, Dentinger B, Diéguez-Uribeondo J, Divakar PK, Douglas B, Dueñas M, Duong TA, Eberhardt U, Edwards JE, Elshahed MS, Fliegerova K, Furtado M, García MA, Ge Z, Griffith GW, Griffiths K, Groenewald JZ, Groenewald M, Grube M, Gryzenhout M, Guo L, Hagen F, Hambleton S, Hamelin RC, Hansen K, Harrold P, Heller G, Herrera C, Hirayama K, Hirooka Y, Ho H, Hoffmann K, Hofstetter V, Högnabba F, Hollingsworth PM, Hong S, Hosaka K, Houbraken J, Hughes K, Huhtinen S, Hyde KD, James T, Johnson EM, Johnson JE, Johnston PR, Jones EG, Kelly LJ, Kirk PM, Knapp DG, Kõljalg U, Kovács GM, Kurtzman CP, Landvik S, Leavitt SD, Liggenstoffer AS, Liimatainen K, Lombard L, Luangsa-ard JJ, Lumbsch HT, Maganti H, Maharachchikumbura SSN, Martin MP, May TW, McTaggart AR, Methven AS, Meyer W, Moncalvo J, Mongkolsamrit S, Nagy LG, Nilsson RH, Niskanen T, Nyilasi I, Okada G, Okane I, Olariaga I, Otte J, Papp T, Park D, Petkovits T, Pino-Bodas R, Quaedvlieg W, Raja HA, Redecker D, Rintoul TL, Ruibal C, Sarmiento-Ramírez JM, Schmitt I, Schüßler A, Shearer C, Sotome K, Stefani FO, Stenroos S, Stielow B, Stockinger H, Suetrong S, Suh S, Sung G, Suzuki M, Tanaka K, Tedersoo L, Telleria MT, Tretter E, Untereiner WA, Urbina H, Vágvölgyi C, Vialle A, Vu TD, Walther G, Wang Q, Wang Y, Weir BS, Weiß M, White MM, Xu J, Yahr R, Yang ZL, Yurkov A, Zamora J, Zhang N, Zhuang W, Schindel D (2012) Nuclear ribosomal internal transcribed spacer (ITS) region as a universal DNA barcode marker for *Fungi*. Proceedings of the National Academy of Sciences of the United States of America 109(16): 6241–6246. 10.1073/pnas.1117018109 PMC3341068 22454494

[B33] Shao MW (2015) Indentification and Metabolites of Symbiotic *Fungi* Isolated from the Gut of Two Kinds of Dragonflies. Zhejiang Normal University.

[B34] Stamatakis A (2014) RAxML version 8 : a tool for phylogenetic analysis and post-analysis of large phylogenies. Bioinformatics 30(9): 1312–1313. 10.1093/bioinformatics/btu033 PMC3998144 24451623

[B35] Sun JL, Zhang HJ, Ding WJ, Zheng DQ, Wang PM, Xu JZ (2020) New azaphilone, isocoumarin and a-pyronederivatives from the marine-derived gut fungus *Paraphaeosphaeria* sp. XZD2-1. Phytochemistry Letters 36: 1–6. 10.1016/j.phytol.2019.12.010

[B36] Tennakoon DS, de Silva NI, Hongsanan S, Xie N (2025) Additions to *Acrocalymmaceae* and *Didymosphaeriaceae* (*Pleosporales*, *Dothideomycetes*): Some interesting novel additions from plant litter in China. MycoKeys 122: 59–98. https://doi.org/10.1016/j.mycokeys.2025.01.001 10.3897/mycokeys.122.163383 PMC12432524 40951763

[B37] Thambugala KM, Wanasinghe DN, Phillips AJL, Camporesi E, Bulgakov TS, Phukhamsakda C, Ariyawansa HA, Goonasekara ID, Phookamsak R, Dissanayake AJ, Tennakoon DS, Tibpromma S, Chen YY, Liu ZY, Hyde KD (2017) Mycosphere notes 1–50: Grass (*Poaceae*) inhabiting *Dothideomycetes*. Mycosphere 8(4): 697–796. 10.5943/mycosphere/8/4/13

[B38] Thippawan T, Lorenzo L, Johannes ZG, Ratchadawan C, Chaiwat TA, Acelino CA, Pedro WC (2014) Mycoparasitic species of *Sphaerellopsis*, and allied lichenicolous and other genera. IMA Fungus 5(2): 391–414. 10.5598/imafungus.2014.05.02.05 PMC4329322 25734030

[B39] Tian XG, Bao DF, Karunarathna SC, Jayawardena RS, Hyde KD, Elgorban AM, Al-Rejaie S, Liu JK (2024) Taxonomy and phylogeny of ascomycetes associated with selected economically important monocotyledons in China and Thailand. Mycosphere 15(1): 1–274. 10.5943/mycosphere/15/1/1

[B40] Vaidya G, Lohman DJ, Meier R (2011) SequenceMatrix: concatenation software for the fast assembly of multi-gene datasets with character set and codon information. Cladistics 27(2): 171–180. 10.1111/j.1096-0031.2010.00329.x 34875773

[B41] Verkley GJM, Dukik K, Renfurm R, Göker M, Stielow JB (2014) Novel genera and species of coniothyrium-like fungi in *Montagnulaceae (Ascomycota)*. Persoonia 32(1): 25–51. 10.3767/003158514X679191 PMC4150078 25264382

[B42] Vidal I, Torres-Vargas JA, Sánchez JM, Trigal M, García-Caballero M, Medina MÁ, Quesada AR (2023) Danthron, an anthraquinone isolated from a marine fungus, is a new inhibitor of angiogenesis exhibiting interesting antitumor and antioxidant properties. Antioxidants 12(5): 1101. 10.3390/antiox12051101 PMC10215650 37237967

[B43] Vilgalys R, Hester M (1990) Rapid genetic identification and mapping of enzymatically amplified ribosomal DNA from several *Cryptococcus* species. Journal of Bacteriology 172(8): 4238–4246. 10.1128/jb.172.8.4238-4246.1990 PMC213247 2376561

[B44] Voglmayr H, Gardiennet A, Jaklitsch WM (2016) *Asterodiscus* and *Stigmatodiscus*, two new apothecial dothideomycete genera and the new order *Stigmatodiscales*. Fungal Diversity 80: 271–284. 10.1007/s13225-016-0356-y PMC5075026 27818618

[B45] Wanasinghe DN, Phukhamsakda C, Hyde KD, Jeewon R, Lee HB, Jones EBG, Tibpromma S, Tennakoon DS, Dissanayake AJ, Jayasiri SC, Gafforov Y, Camporesi E, Bulgakov TS, Ekanayake AH, Perera RH, Samarakoon MC, Goonasekara ID, Mapook A, Li WJ, Senanayake IC, Li JF, Norphanphoun C, Chaiwan N, Dong Y, Jayawardena RS, Perera J, de Silva NI, Ti O, Zhang W (2018) Fungal diversity notes 709–839: taxonomic and phylogenetic contributions to fungal taxa with an emphasis on fungi on *Rosaceae*. Fungal Diversity 89: 1–236. 10.1007/s13225-018-0395-7

[B46] Wanasinghe DN, Phookamsak R, Dissanayake LS, Xu J (2025) Unexplored microfungal diversity from dead woody litter in the dry-hot valleys of Honghe (Yunnan, China). Studies in Fungi 10: e017. 10.48130/sif-0025-0016

[B47] White TJ, Bruns TD, Lee SB, Taylor JW (1990) Amplification and direct sequencing of fungal ribosomal RNA genes for phylogenetics. PCR protocols: a guide to methods and applications 18(1): 315–322. 10.1016/B978-0-12-372180-8.50042-1

[B48] Wong MKM, Goh TK, Hyde KD (2000) *Paraphaeosphaeria schoenoplecti* sp. nov. on senescent culms of *Schoenoplectus litoralis* in Hong Kong. Fungal Diversity 4: 171–179.

