# Supplementary material for: Additions to Didymosphaeriaceae: two novel species of Chromolaenicola crataegicola and Paraphaeosphaeria fulva with notes on antibacterial and antifungal activities
Source: MycoKeys. 2026 Jul 20;137:43–67. doi: 10.3897/mycokeys.137.197035 (PMC13408569; doi:10.3897/mycokeys.137.197035)
Supplement: Supplementary material 1 — Supplementary images [file mycokeys-137-043-s001.docx]

**SUPPLEMENTARY MATERIAL FOR**

**Additions to Didymosphaeriaceae: two novel species of *Chromolaenicola* and *Paraphaeosphaeria* with notes on antibacterial and antifungal activities**

**Figure S1:** The best-scoring RAxML tree based on a concatenated ITS dataset of Didymosphaeriaceae.

**Figure S2:** The best-scoring RAxML tree based on a concatenated LSU dataset of Didymosphaeriaceae.

**Figure S3:** The best-scoring RAxML tree based on a concatenated SSU datasetof Didymosphaeriaceae.


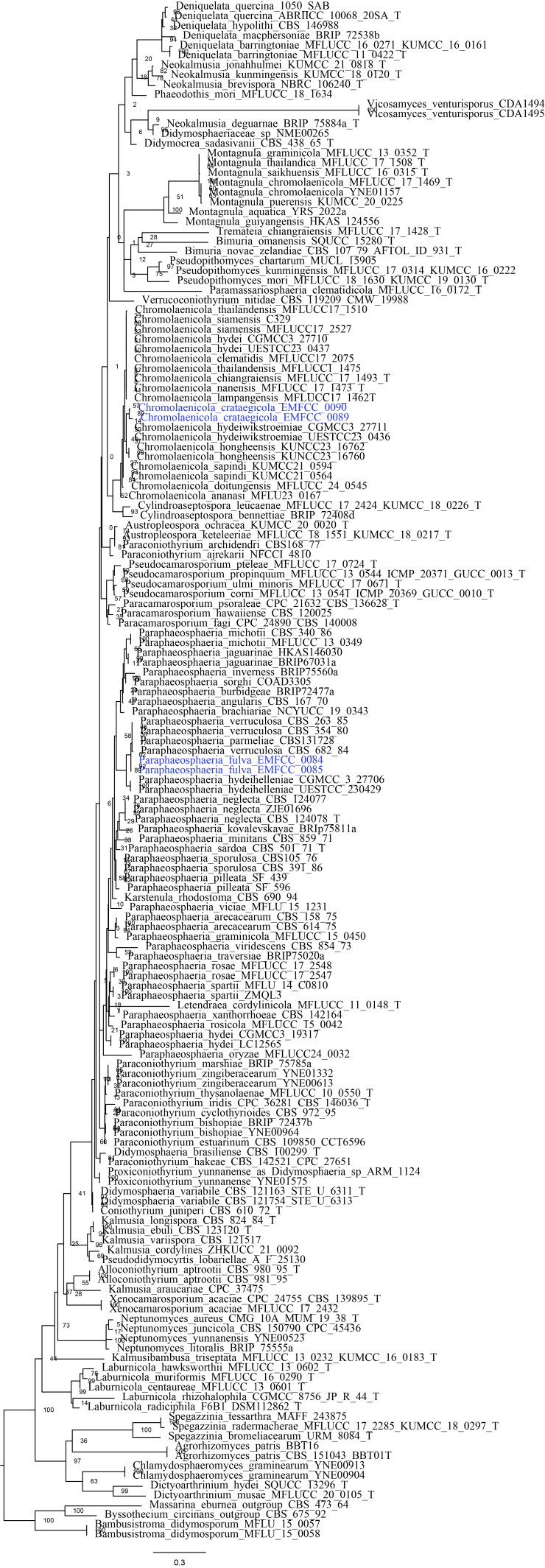


Figure S1: The best-scoring RAxML tree based on a concatenated ITS dataset of Didymosphaeriaceae.


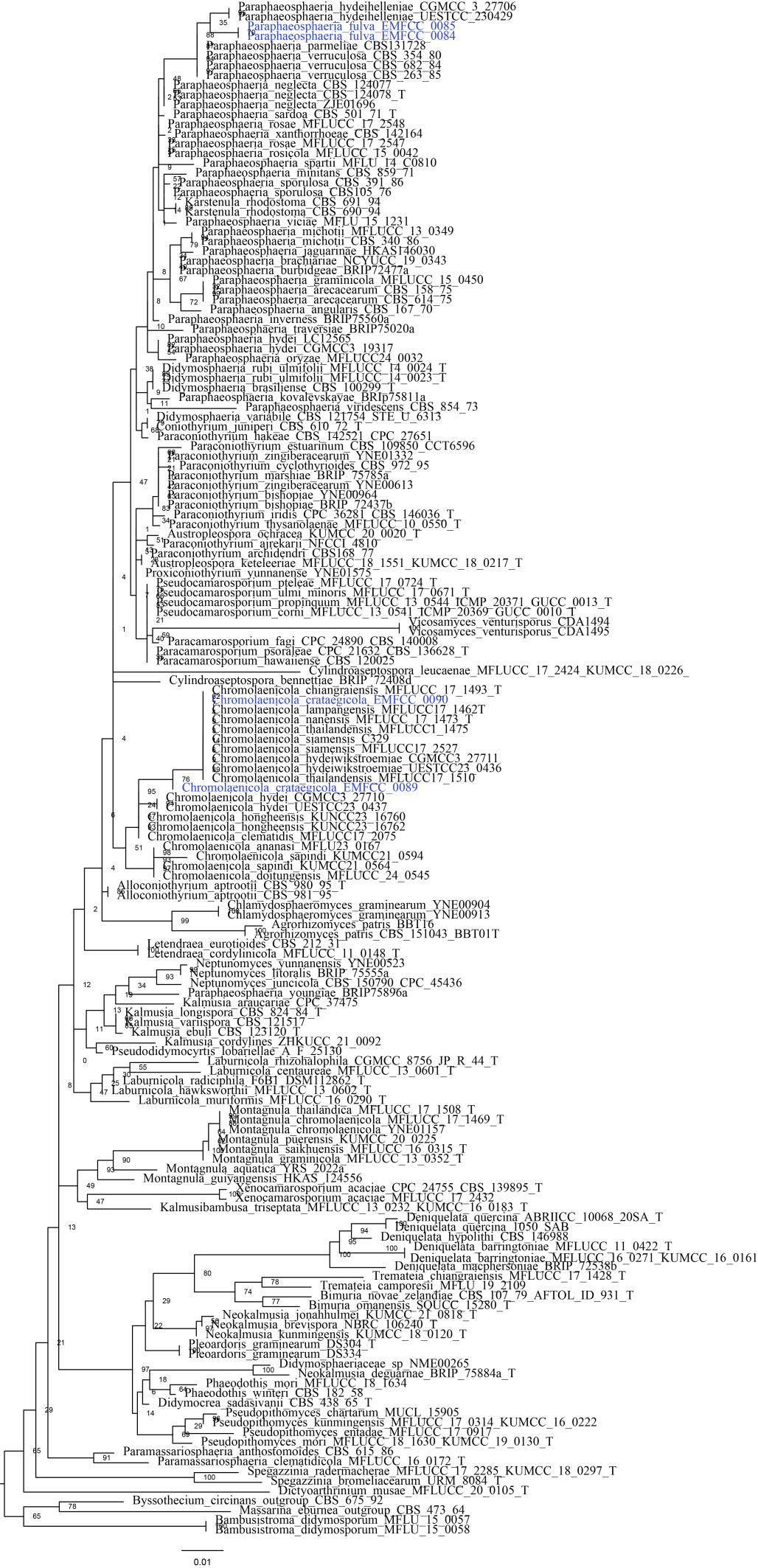


Figure S2: The best-scoring RAxML tree based on a concatenated LSU dataset of Didymosphaeriaceae.


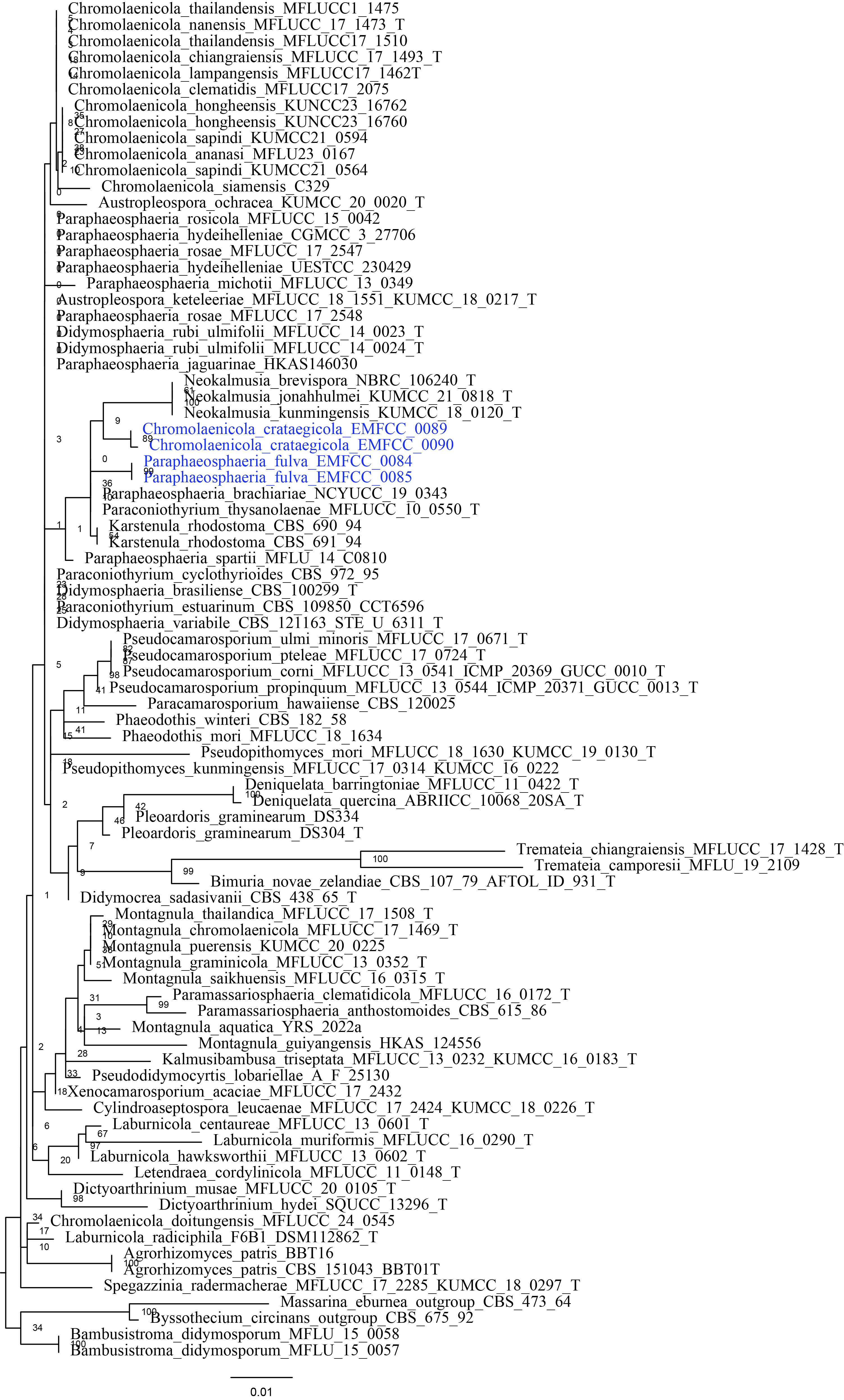


Figure S3: The best-scoring RAxML tree based on a concatenated SSU datasetof Didymosphaeriaceae.
